# QTL Landscape for Oil Content in *Brassica juncea*: Analysis in Multiple Bi-Parental Populations in High and “0” Erucic Background

**DOI:** 10.3389/fpls.2018.01448

**Published:** 2018-10-16

**Authors:** Kadambini Rout, Bal Govind Yadav, Satish Kumar Yadava, Arundhati Mukhopadhyay, Vibha Gupta, Deepak Pental, Akshay K. Pradhan

**Affiliations:** ^1^Department of Genetics, University of Delhi South Campus, New Delhi, India; ^2^Centre for Genetic Manipulation of Crop Plants, University of Delhi South Campus, New Delhi, India

**Keywords:** *Brassica juncea*, seed oil content, seed erucic acid content, QTL mapping, meta-analysis, pleiotropy

## Abstract

Increasing oil content in oilseed mustard (*Brassica juncea*) is a major breeding objective—more so, in the lines that have “0” erucic acid content (< 2% of the seed oil) as earlier studies have shown negative pleiotropic effect of erucic acid loci on the oil content, both in oilseed mustard and rapeseed. We report here QTL analysis of oil content in eight different mapping populations involving seven different parents—including a high oil content line J8 (~49%). The parental lines of the mapping populations contained wide variation in oil content and erucic acid content. The eight mapping populations were categorized into two sets—five populations with individuals segregating for erucic acid (SE populations) and the remaining three with zero erucic acid segregants (ZE populations). Meta-analysis of QTL mapped in individual SE populations identified nine significant *C-QTL*, with two of these merging most of the major oil QTL that colocalized with the erucic acid loci on the linkage groups A08 and B07. QTL analysis of oil content in ZE populations revealed a change in the landscape of the oil QTL compared to the SE populations, in terms of altered allelic effects and phenotypic variance explained by ZE QTL at the “common” QTL and observation of “novel” QTL in the ZE background. The important loci contributing to oil content variation, identified in the present study could be used in the breeding programmes for increasing the oil content in high erucic and “0” erucic backgrounds.

## Introduction

Oilseed mustard, *Brassica juncea* (AABB), is an important oilseed crop of the south Asian region. In India alone, the crop is grown on around six million hectares of land (Chauhan et al., 2011) under low moisture regimes during the winter growing season. Breeding for high oil content and improved oil quality are two key breeding objectives in oilseed mustard. The quality of oil in *Brassica* species, including mustard, is determined by the relative proportions of different fatty acid fractions—saturated fatty acids such as palmitic (C16:0), stearic (C18:0) and unsaturated fractions such as oleic (C18:1), eicosenoic (C20:1), erucic (C22:1), linoleic (C18:2), and linolenic acid (C18:3). Erucic acid (C22:1), a long-chain monounsaturated fatty acid is a major fraction (~50%) of the seed oil in *Brassica* species. Presence of high erucic acid content has been shown to be anti-nutritional (Ackman et al., 1977). Breeding for “0” erucic acid content (< 2% of the seed oil) has been a major breeding objective in mustard. Almost all the pureline varieties and hybrids of rapeseed (*Brassica napus*, AACC) grown in north America, Europe, China, and many other parts of the world are “0” erucic (ZE). However, development of productive ZE mustard is still an unachieved objective even though experimental lines with “0” erucic content have been available for quite some time (Kirk and Oram, 1981). One of the major reasons for the low acceptability of ZE mustard is its lower seed oil content as compared to high erucic (HE) lines.

We had earlier mapped the fatty acid elongase (*FAE1*) genes involved in erucic acid biosynthesis in *B. juncea* to LGs A08 (*FAE1.1)* and B07 (*FAE1.2)* by using a F_1_DH mapping population derived from a cross of an Indian gene pool line Varuna (HE) with an exotic line Heera (ZE) (Gupta et al., 2004). Transfer of these two ZE loci to the extensively grown mega-variety Varuna through marker-assisted backcross breeding led to the development of ZE-Varuna; however, the oil content in ZE-Varuna was reduced to ~36% as compared to ~43% in HE-Varuna (Jagannath et al., 2011). Many other studies have also reported such a kind of negative association between erucic acid content and overall seed oil content, suggesting a negative pleiotropic effect of ZE trait on the oil content both in rapeseed (Ecke et al., 1995; Burns et al., 2003) and mustard (Mahmood et al., 2006; Jagannath et al., 2011). Breeding for high oil content concomitant with ZE trait would require identification of compensatory loci that can contribute to high oil content in ZE background (Pradhan and Pental, 2011).

A number of studies have mapped QTL for seed oil content in mustard (Cheung et al., 1998; Mahmood et al., 2006; Ramchiary et al., 2007; Yadava et al., 2012) and rapeseed (Ecke et al., 1995; Delourme et al., 2006; Qiu et al., 2006; Cao et al., 2010; Sun et al., 2012; Zhao et al., 2012; Wang et al., 2013; Jiang et al., 2014; Fu et al., 2017). Most of the studies have used a single bi-parental population with small parental difference for the oil content. Only two studies in *B. napus*, Sun et al. (2012) and Wang et al. (2013), have used parents with more than 10% difference in oil content. Recently, Fu et al. (2017) undertook QTL analysis of oil content by association mapping using 142 rapeseed breeding lines with diverse oil content ranging from 37.39 to 55.28%. However, none of these studies have identified and highlighted QTL that would be useful in breeding for oil content in ZE background. We have earlier mapped QTL for oil content in two bi-parental populations derived from the F_1_ of a cross between Varuna and Heera, one subset segregating for erucic acid loci (SE population) and the other subset consisting only of ZE individuals (Jagannath et al., 2011). However, for broader MAS (marker-assisted selection) application, consensus QTL identified through consolidation of QTL data from multiple bi-parental mapping populations by a joint and integrative analysis would be more pertinent than the QTL identified in a single bi-parental population. Moreover, QTL data derived from multiple populations with wider genetic variability for the trait is more informative than using a single bi-parental population with large parental contrast as the former would allow identification of more number of alleles with their effects and assist breeders in marker assisted transfer of the trait more effectively.

Therefore, a major objective of the present study was to define the QTL involved in seed oil content variation in *B. juncea* using multiple bi-parental mapping populations. Another objective was to identify specific loci operative in a ZE acid background. The parents used in the study encapsulate maximum genetic variability for the seed oil content in *B. juncea*. Through the use of two sets of bi-parental mapping populations—one set segregating for erucic acid (named as SE populations) and the other set consisting of zero erucic acid segregants (named as ZE populations), the QTL analysis identified pleiotropic QTL for seed oil content in *B. juncea*. The study also revealed a change in the genetic landscape of QTL and identified “novel” QTL in ZE populations which otherwise remained masked due to the influence of erucic acid in the SE populations. Based on a detailed genetic analysis of seed oil content, a relevant breeding strategy for the enhancement of oil content in ZE mustard has been discussed.

## Materials and methods

### Plant materials

Five bi-parental F_1_DH mapping populations derived from seven purelines of *B. juncea* were used in the study. The seven parental lines contained wide variation for both seed oil content and erucic acid, including a pureline J8 with an oil content of ~49%—highest reported so far in oilseed mustard. The details of these parental lines are given in Table 1. These mapping populations were developed by microspore culture following an established protocol (Mukhopadhyay et al., 2007). Eight different mapping populations were sourced from these five bi-parental source DH populations (Table 2). Of these eight populations, five populations consisted of individuals segregating for the erucic acid trait (called SE populations) and were named as EJ8^A8B7^, DE^B7^, EPJ^A8B7^, TD^A8^, and VH^A8B7^, the remaining three mapping populations consisted of only “0” erucic acid segregants (called ZE populations) and were named as EJ8^Z^, DE^Z^, and VH^Z^ (Table 2). As all the five F_1_DH source populations segregate for one or both the erucic acid genes, five SE populations were constructed (EJ8^A8B7^, DE^B7^, EPJ^A8B7^, TD^A8^, and VH^A8B7^) from their respective F_1_DH source population by random sampling. On the other hand, for the construction of three ZE populations (EJ8^Z^, DE^Z^, and VH^Z^), the selfed seeds of respective F_1_DH source population were initially analyzed by GC to identify the zero erucic acid segregants and the mapping populations were constructed by random sampling from these zero erucic acid segregants. Detailed genesis of these populations is shown in Supplementary Figure 1. Genotype of parental lines for two erucic acid genes in LG A08 and B07 was ascertained by *FAE1* gene markers following the protocol of Gupta et al. (2004).

**Table 1:** Phenotypic variation for oil content and erucic acid in seven parental lines used for developing the five bi-parental mapping populations.

**Parental line**	**Oil content (%)**	**Erucic acid content (%)**	**Genotype at the erucic acid (*****FAE1*****) locus**^**a**^		**Description**
			**LG A08**	**LG B07**	
J8	49.3 ± 0.6	55.5 ± 1.2	*E1E1*	*E2E2*	A recombinant inbred line derived from Indian and east European germplasm with the highest oil content reported so far
Pusa Jaikisan	41.1 ± 2.0	55.3 ± 2.0	*E1E1*	*E2E2*	A bold seeded Indian gene pool variety
Varuna	42.6 ± 2.3	47.3 ± 2.3	*E1E1*	*E2E2*	A widely grown mega variety of mustard
TM-4	40.7 ± 0.5	52.8 ± 3.8	*E1E1*	*E2E2*	A pureline developed by mutagenesis
Donskaja-IV	44.6 ± 1.5	20.5 ± 2.5	*e1e1*	*E2E2*	A representative line of the east European gene pool
Heera	39.0 ± 1.3	0.0	*e1e1*	*e2e2*	A “00” east European mustard line
EH-2	36.9 ± 1.1	0.0	*e1e1*	*e2e2*	An early maturing derivative of Heera

a *Genotype of two erucic acid loci in LG A08 and B07 was ascertained by single nucleotide polymorphism reported in the candidate genes FAE1.1 and FAE1.2, respectively (Gupta et al., 2004)*.

**Table 2:** Phenotypic performance of the parents and eight bi-parental mapping populations of *B. juncea* for seed oil content at different locations.

**Mapping population^a^**	**Size of the source DH population**	**Size of the mapping population**	**Environment**	**Parental mean**		**Range and mean of mapping population**		**Broad sense heritability (%)**
				**Parent 1^b^**	**Parent 2^b^**	**Range**	**Mean ±SD**	
**SE MAPPING POPULATIONS**								
EJ8^A8B7^	1000	166	Alwar	48.9	37.0	36.4–51.0	45.3 ± 2.7	84.0
			Bharatpur	49.1	37.6	38.1–50.5	44.6 ± 2.4	52.0
			Delhi	50.0	36.9	34.1–49.3	43.8 ± 2.8	84.0
EPJ^A8B7^	654	182	Alwar	39.3	38.6	28.6–47.8	40.5 ± 3.1	66.0
			Bharatpur	43.2	38.3	29.1–45.7	40.5 ± 2.6	69.0
			Delhi	40.8	35.6	30.0–44.3	39.2 ± 2.4	86.0
VH^A8B7^	1200	123	Delhi	43.9	39.6	29.5–46.2	39.2 ± 3.3	75.0
			Gwalior	44.0	40.0	37.9–50.6	44.1 ± 2.6	68.0
			Leh	39.9	37.5	35.7–48.5	41.6 ± 2.3	69.0
DE^B7^	296	163	Delhi Year 1	43.1	35.7	30.3–46.7	40.0 ± 2.9	67.0
			Delhi Year 2	44.2	36.1	29.0–44.6	38.8 ± 2.9	61.0
			Delhi Year 3	43.5	35.9	32.0−47.5	40.2 ± 3.0	64.0
TD^A8^	143	100	Delhi	41.2	46.7	32.8–45.2	39.9 ± 2.6	74.0
			Bharatpur	40.2	–	26.5–42.3	35.2 ± 3.2	74.0
			Leh	40.6	45.4	39.8–52.9	46.1 ± 2.4	83.0
**ZE MAPPING POPULATIONS**								
EJ8^Z^	1000	166	Alwar	46.8	36.4	33.3–42.1	38.5 ± 1.7	51.0
			Bharatpur	39.1	33.1	27.5–36.3	32.0 ± 1.7	41.0
			Delhi	50.0	38.4	35.8–44.9	40.2 ± 1.7	51.0
DE^Z^	296	54	Delhi Year 1	43.1	35.7	30.4–42.0	38.1 ± 2.4	78.0
			Delhi Year 2	44.2	36.1	29.0–42.7	37.3 ± 2.9	85.0
			Delhi Year 3	43.5	35.9	32.0–43.9	38.5 ± 2.8	82.0
VH^Z^	1200	110	Delhi	43.9	39.6	25.0–42.1	32.0 ± 3.2	75.0

a *The superscripts in different SE populations indicate the segregation status of the two erucic acid genes on LGs A08 and B07 and superscript “Z” indicates the populations with “0” erucic acid segregants*.

b *Parents used for development of the eight bi-parental mapping populations*.

**Table:** 

*Mapping population*	*Parent1*	*Parent2*
*EJ8^A8B7/Z^*	*J8*	*EH-2*
*DE^B7/Z^*	*Donskaja-IV*	*EH-2*
*EPJ^A8B7^*	*Pusa Jaikisan*	*EH-2*
*TD^A8^*	*TM-4*	*Donskaja-IV*
*VH^A8B7/Z^*	*Varuna*	*Heera*

### Field experiments and trait measurements

The oil content and erucic acid content data for EJ8^A8B7/Z^, DE^B7/Z^, and EPJ^A8B7^ populations were estimated from the seeds by growing all the bi-parental mapping populations either in three different locations or a single location over three years during the mustard growing season (Table 2). A randomized block design with three replications for each entry was adopted for field experiments of all the mapping populations. Each one of the mapping population and their respective parental lines were planted in three rows with a row length of 3 m per replication. On maturity, open pollinated seeds of five competitive plants from the middle row of each replication were pooled, desiccated and used for the estimation of the seed oil content (expressed in percentage) by NIRS (Mika et al., 2003). Erucic acid content was estimated by GC using a Perkin Elmer AutosystemXL GC with an Elite-FFAP column (3 mm length × 0.25 mm ID; 0.25 μm thick film) from the selfed seeds following an established protocol (Thies, 1971). The trait data for VH^A8B7/Z^ and TD^A8^ populations were retrieved from earlier reports (Gupta et al., 2004; Ramchiary et al., 2007; Jagannath et al., 2011; Yadava et al., 2012). All the estimations were done in triplicate and the mean value was used as the trait value.

### Linkage map construction and QTL analysis

For QTL mapping, eight bi-parental linkage maps were developed using the five SE and three ZE mapping populations (Table 3). Genotyping of the mapping populations was done by using various marker systems, such as IP (Intron Polymorphism) (Panjabi et al., 2008), genic SSRs (Dhaka et al., 2017), genomic SSRs (Padmaja et al., 2005; Kim et al., 2009; Xu et al., 2010), SNPs (Paritosh et al., 2014), RFLP and AFLP markers (Pradhan et al., 2003). The linkage maps were constructed using JoinMap 4.0 (Van Ooijen, 2006) following the parameters used by Pradhan et al. (2003). The linkage groups were established with a minimum LOD threshold of 7.0, and the Kosambi function was used to convert recombination frequencies to cM distances. Of the eight bi-parental maps used in the study, three maps namely, EJ8^A8B7^, EJ8^Z^, and DE^Z^ were constructed in the present study (Table 3). EJ8^A8B7^ map consists of 388 markers and covers a total map length of 1125.6 cM. EJ8^Z^ and DE^Z^ maps consist of 470 and 650 markers and cover map lengths of 1244.0 and 1680.2 cM, respectively. The remaining five bi-parental maps that have been reported earlier (Table 3), were updated with some SSR markers to enhance the genome coverage. Detailed map characteristics of all the eight bi-parental linkage maps used for the QTL analysis are given in Table 3.

**Table 3:** Summary of the five SE and three ZE bi-parental genetic maps.

**Name of the map**	**Details of mapped markers^a^**						**Map characteristics**					**Comments**
	**IP**	**Genic SSR**	**Genomic SSR**	**SNP**	**AFLP**	**RFLP**	**Total**	**Map length (cM)**	**Average interval size (cM)**	**Marker density**	**No. of common markers^b^**	
**SE MAPS**^c^												
EJ8^A8B7^	199	47	30	112	0	0	388	1125.6	3.9	0.3	240	Developed in the present study
EPJ^A8B7^	233	454	152	2	0	0	841	2055.2	3.1	0.4	390	Reconstructed the EPJ map reported by Dhaka et al. (2017) using 2 SNP markers and removing 21 redundant markers
VH^A8B7^	704	83	267	954	389	11	2408	1865.1	1.1	1.3	–	Reconstructed a consolidated map by combining the earlier reported VH maps (Ramchiary et al., 2007; Panjabi et al., 2008; Paritosh et al., 2014; Rout et al., 2015) and addition of 89 SSR markers
DE^B7^	342	94	192	25	0	0	653	1708.2	4.3	0.4	256	Reconstructed the DE map reported by Rout et al. (2015) adding 8 SSR markers and removing 8 redundant markers
TD^A8^	298	0	73	1	509	0	881	1565.5	2.5	0.6	420	Updated the TD map reported by Yadava et al. (2012) with 65 SSR markers and the FAE1.1 gene marker
**ZE MAPS**^c^												
EJ8^Z^	162	138	10	160	0	0	470	1244.0	3.2	0.4	271	Developed in the present study
DE^Z^	341	95	190	24	0	0	650	1680.2	4.8	0.4	254	Developed in the present study
VH^Z^	10	0	2	0	408	0	420	1614.0	3.8	0.3	234	Jagannath et al., 2011

a *Sources of different marker systems used in the present study are given in the Supplementary Table 4*.

b *Indicates the number of markers common between the individual map and the reference map (VH^A8B7^) used for QTL meta-analysis*.

c *The superscripts in different SE populations indicate the segregation status of two erucic acid genes on LGs A08 and B07 and superscript “Z” indicates the populations with “0” erucic acid segregants*.

Composite interval mapping (CIM) in Windows QTL Cartographer 2.5 (Wang et al., 2012), with a window size of 10 cM and a walk speed of 1 cM with five control markers, was performed for QTL analysis. For declaring the presence of a QTL, genome-wide threshold values (*P* = 0.05) were estimated from 1,000 permutations of trait data across all genetic intervals (Churchill and Doerge, 1994; Doerge and Churchill, 1996). QTL detected with more than 10% of the phenotypic variance (*R*^2^) were considered as major QTL. In addition, to correlate structures from among the two quantitative traits—erucic acid and oil content in seeds, we used multiple-trait mapping (Mt-CIM) implemented in Windows QTL Cartographer 2.5 to evaluate the pairs of variables jointly (Jiang and Zeng, 1995). This analysis was based on composite interval mapping similar to the procedure adopted for QTL analysis of single traits.

QTL meta-analysis was performed using BioMercator 2.1 (Arcade et al., 2004). Initially, one integrated map (INT map) was developed incorporating the eight bi-parental maps through iterative projection of genetic maps of both the SE and ZE populations on to the VH^A8B7^ map (reference map) using the ‘Map projection' command. The markers mapped on the integrated map were assigned to the *Arabidopsis* genomic blocks (Schranz et al., 2006) based on their similarity with *B. rapa* genes available in the Brassica database (http://brassicadb.org/brad), as obtained through BLASTN search.

For QTL meta-analysis, the identified QTL in the respective populations were projected on to the INT map and consensus QTL were discerned using a two-step approach. In the first step, QTL obtained from the five SE populations were integrated to identify consensus QTL that included the merged QTL from different mapping populations into a single QTL and was named as a *C-QTL*. Those QTL which did not merge were designated as “population-specific” QTL. In the second step, QTL identified from both SE and ZE populations were pooled together to identify the “common” QTL between the SE and ZE populations. The name of “common” QTL started with the trait name followed by LG name and the serial number of the QTL on that LG (e.g., *Oil-A2-1*).

The allelic effects of parental lines at these QTL merging into a *C-QTL* and “common” QTL were estimated following the procedure of Chardon et al. (2014), followed by elucidation of the allelic status of the parental lines using Heera/EH-2 as the common parent (Rout et al., 2015).

## Results

### Trait variation in parents and mapping populations

Trait values for oil content and erucic acid content among the seven parental lines used in the study for genetic mapping of oil content trait are given in Table 1. These parental lines covered maximum phenotypic variability reported for the two traits in *B. juncea*. The phenotypic variation for the oil content ranged from ~36.9% in EH-2 to ~49.3% in J8. For the erucic acid content, two parents Heera and EH-2, both ZE lines, harbor the recessive alleles for the two erucic acid genes on the LGs A08 and B07. The lines, Varuna, J8, Pusa Jaikisan and TM-4 with high erucic acid content (>47.0%) contained both the dominant alleles for high erucic acid content on the LGs A08 and B07. The parental line, Donskaja-IV with intermediate levels of erucic acid (~20.5%) is homozygous dominant for the LG B07 locus and homozygous recessive for the LG A08 locus of the erucic acid trait (Table 1).

Frequency distributions of oil content showed continuous variation in all the eight mapping populations in all the locations, signifying the quantitative nature of the trait (Supplementary Figure 2). Transgressive segregation in both the directions was apparent in four of the five SE populations (VH^A8B7^, EPJ^A8B7^, DE^B7^, and TD^A8^) whereas in the EJ8^A8B7^ population, transgressive variation beyond the high value parent was not observed. It can be concluded that the parental line J8 with the highest trait value has accumulated maximum number of positive alleles for oil content. A very high positive correlation (significant at 1% level) between erucic acid content and seed oil content was observed in the five SE populations in all the environments (Supplementary Table 1). The correlation coefficients ranged from 0.77 in EJ^A8B7^ population to 0.39 in DE^B7^ population.

All the three ZE populations showed reduction in their mean oil content as compared to the respective SE populations (Table 2). ZE populations did not show any transgressive segregation for oil content beyond the contents in the high value parents in all the environments (Supplementary Figure 2).

### QTL analysis of seed erucic acid content (*Eru*) in SE populations

QTL analysis of erucic acid content in EJ8^A8B7^, EPJ^A8B7^, and VH^A8B7^ populations, identified major QTL on the LGs A08 and B07 in all the three environments co-mapping to the two *FAE1* loci (Supplementary Table 2). The average phenotypic variance (*R*^2^) explained by these QTL in these three populations varied from 87.5% (in EPJ^A8B7^ population) to 94.0% (in VH^A8B7^ population). A few minor loci explaining 1.0 to 2.1% of phenotypic variance were also identified in the EJ8^A8B7^and EPJ^A8B7^ populations.

QTL analysis of erucic acid in the other two SE populations (DE^B7^ and TD^A8^) wherein only one locus was segregating for erucic acid, detected major QTL for the trait in all the environments co-mapping with the respective *FAE1* locus. In DE^B7^ population segregating for erucic acid on LG B07, the average phenotypic variance explained by the major QTL was 90.7% whereas in the TD^A8^ population segregating for erucic acid on LG A08, the average phenotypic variance explained by the major QTL was 46.6%. In addition, in both the populations some minor QTL were also detected (Supplementary Table 2).

### QTL analysis of seed oil content (*oil*) in SE populations

Mapping of the oil content in the EJ8^A8B7^ population showing maximum parental contrast for the oil content, detected a total of 15 *Oil* QTL from three environments (Supplementary Table 3). These QTL were distributed over two A sub-genome LGs (A07 and A08) and six B sub-genome LGs (B01, B02, B03, B04, B06, and B07). The phenotypic variance (*R*^2^) explained by the individual QTL varied from 2.5 to 43.3% with additive effects ranging from 0.4 to 1.8%. Six QTL were detected as major effect QTL, three each on LGs A08 (mean *R*^2^ = 34.0%) and B07 (mean *R*^2^ = 12.9%), all co-mapping with the two *FAE1* loci on the corresponding LGs—A08 and B07. Remaining nine QTL were detected as minor QTL with phenotypic variance explained ranging from 2.5 to 7.7%. All the QTL, except *Oil-B2-1-EJ* and *Oil-B2-2-EJ*, had the trait enhancing allele from the high oil parent J8.

The EPJ^A8B7^ population detected the highest number of 23 *Oil* QTL from three environments (Supplementary Table 3). These QTL were distributed over nine LGs, four in the A sub-genome and five in the B sub-genome. The phenotypic variance explained by these 23 *Oil* QTL ranged from 2.6 to 35.2% wherein Pusa Jaikisan and EH-2 contributed the positive alleles at 11 and 12 QTL, respectively. Of these 23 QTL, three QTL that co-mapped to the erucic acid QTL region on LG A08 were detected as major effect QTL (mean *R*^2^ = 33.2%). All other QTL including the three QTL in erucic acid region on LG B07 were detected as minor QTL.

The *Oil* QTL analysis in VH^A8B7^ population detected a total of 12 QTL of which eight QTL were detected on LGs A07, A08, A09, and A10, and four QTL on the LGs B07 and B08 (Supplementary Table 3). Eight QTL had trait enhancing allele from the high-value parent Varuna and four QTL from the low-value parent Heera. Five of the seven major QTL were co-mapped to the erucic acid QTL regions on the LGs A08 and B07.

The DE^B7^ population, segregating only for the erucic acid locus on LG B07 (Table 1), identified a total of 13 *Oil* QTL distributed over eight linkage groups, four LGs each of the A and B sub-genomes (Supplementary Table 3). The phenotypic variance explained by these QTL ranged from 4.7 to 23.0%. In six of the 13 QTL, the positive allele was contributed by Donskaja-IV, whereas EH-2 contributed the remaining seven QTL. Three major QTL were detected on LG B07, all of which co-mapped to the erucic acid QTL region. On LG A08, a minor QTL (*Oil-A8-1-DE*) was detected from a single location that mapped at a distance of ~25 cM from the position of the *FAE1.1* gene.

The TD^A8^ population showing segregation for erucic acid locus on LG A08 had dominant allele in both the parents for the erucic acid locus on LG B07 (Table 1). QTL analysis identified nine *Oil* QTL from three environments (Supplementary Table 3). These QTL were distributed over three LGs of the A sub-genome and four LGs of the B sub-genome. The phenotypic variance explained by the QTL ranged from 7.2 to 22.4%. Of the nine QTL detected in the population, seven had acquired the positive allele from TM-4, while two had positive allele from Donskaja-IV. Six QTL were detected as major QTL, of which two QTL overlapped with the erucic acid QTL containing the erucic acid gene on LG A08 (Supplementary Table 3).

*Oil* QTL analysis from five bi-parental SE populations identified a total of 72 QTL, of which 40 were derived from the parental lines with high trait value, and the remaining 32 had the trait enhancing allele from the low oil content parents. The identified QTL were found to be distributed over 16 of the 18 LGs of *Brassica juncea*, except LGs A06 and B05, with almost equal contribution from both the A and B sub-genomes showing a wide distribution of loci influencing oil content. A total of 25 QTL were detected as major QTL with *R*^2^ greater than 10%, of which 19 QTL overlapped with erucic acid QTL regions including six high effect QTL explaining ≥25% of the phenotypic variance of oil content (Supplementary Table 3).

### Meta-QTL analysis of oil content in SE populations

An integrated (INT) map was developed by merging the markers from eight bi-parental maps and contained a total of 3510 markers including 974 Intron Polymorphism, 917 SNP, 535 genic SSR, 428 genomic SSR, 645 AFLP, and 11 RFLP markers covering a genetic length of 2002.6 cM (Supplementary Table 4). About 70% of the mapped markers could be assigned *Arabidopsis thaliana* gene IDs and the corresponding genomic blocks of *Arabidopsis* (Schranz et al., 2006).

The 72 *Oil* QTL identified from the five SE mapping populations were distributed on the 16 LGs of *B. juncea*. QTL meta-analysis was undertaken only for 65 QTL that were distributed over 12 LGs, while excluding the remaining seven QTL which were detected only in one of the five bi-parental mapping populations. Meta-analysis resulted in nine *C-QTL* (Supplementary Table 4) distributed over nine LGs by merging a total of 47 *Oil* QTL mapped in the individual populations (Table 4, Supplementary Figure 3). The number of component QTL that formed a *C-QTL* varied from two to 12. No *C-QTL* was formed by merging component QTL from all the five SE populations. Seven out of the nine *C-QTL* merged the component QTL from two populations, each. Wherein, the remaining two *C-QTL* were formed by merging component QTL from four populations, each (Table 4).

**Table 4:** Details of consensus QTL (*Oil-C*) detected by merging the QTL data of five SE populations (EJ8^A8B7^, EPJ^A8B7^, VH^A8B7^, DE^B7^, and TD^A8^) through QTL meta-analysis.

**Details of** ***C-QTL***				**Component QTL**		
***C-QTL***	**LG**	**CI (cM)**	**Flanking markers**	**QTL**	***R*^2^ (%)^b^**	**Donor parent**
*Oil-C-A7-1*	A07	45.9–70.1	cnu_m341a–At1g73990	*Oil-A7-1-EJ*	3.3	J8
				*Oil-A7-2-EJ*	6.2	J8
				*Oil-A7-1-VH*	12.5	Heera
				*Oil-A7-2-VH*	8.9	Heera
*Oil-C-A8-1*^a^	A08	7.8–10.2	UGM0947–UGM2062a	*Oil-A8-1-EJ*	28.2	J8
				*Oil-A8-2-EJ*	30.5	J8
				*Oil-A8-3-EJ*	43.3	J8
				*Oil-A8-1-EPJ*	30.1	Pusa Jaikisan
				*Oil-A8-2-EPJ*	34.3	Pusa Jaikisan
				*Oil-A8-3-EPJ*	35.2	Pusa Jaikisan
				*Oil-A8-1-VH*	15.2	Varuna
				*Oil-A8-2-VH*	24.2	Varuna
				*Oil-A8-3-VH*	13.8	Varuna
				*Oil-A8-1-TD*	12.4	TM-4
				*Oil-A8-2-TD*	17.0	TM-4
*Oil-C-A10-1*	A10	6.3–25.2	At1g04950–At5g16480	*Oil-A10-1-VH*	11.3	Varuna
				*Oil-A10-2-VH*	7.3	Varuna
				*Oil-A10-1-TD*	11.8	TM-4
				*Oil-A10-2-TD*	22.4	TM-4
*Oil-C-B1-1*	B01	49.9–89.5	At5g61420c–UGM1780b	*Oil-B1-1-EJ*	2.6	J8
				*Oil-B1-2-EJ*	7.4	J8
				*Oil-B1-1-EPJ*	3.3	Pusa Jaikisan
*Oil-C-B2-1*	B02	66.0–83.0	At4g16280–At4g33925	*Oil-B2-1-EJ*	5.0	EH-2
				*Oil-B2-2-EJ*	7.7	EH-2
				*Oil-B2-1-EPJ*	6.5	EH-2
				*Oil-B2-2-EPJ*	3.4	EH-2
				*Oil-B2-3-EPJ*	3.8	EH-2
*Oil-C-B3-1*	B03	47.5–73.8	UGM1946–BJ_VH_0551	*Oil-B3-1-EJ*	3.8	J8
				*Oil-B3-1-EPJ*	9.4	EH-2
				*Oil-B3-2-EPJ*	9.8	EH-2
				*Oil-B3-3-EPJ*	9.0	EH-2
*Oil-C-B4-1*	B04	43.5–85.2	UGM1548–BJ_VH_0337	*Oil-B4-1-EJ*	3.5	J8
				*Oil-B4-1-DE*	4.7	Donskaja-IV
*Oil-C-B6-1*	B06	31.3–51.2	At1g63980–BJ_VH_0350	*Oil-B6-1-EJ*	2.5	J8
				*Oil-B6-1-DE*	7.6	Donskaja-IV
*Oil-C-B7-1*^a^	B07	50.8–59.3	e48t78v205–e47t70t400	*Oil-B7-1-EJ*	12.2	J8
				*Oil-B7-2-EJ*	13.7	J8
				*Oil-B7-3-EJ*	12.8	J8
				*Oil-B7-1-EPJ*	9.8	Pusa Jaikisan
				*Oil-B7-2-EPJ*	8.1	Pusa Jaikisan
				*Oil-B7-3-EPJ*	9.3	Pusa Jaikisan
				*Oil-B7-1-VH*	12.0	Varuna
				*Oil-B7-2-VH*	9.4	Varuna
				*Oil-B7-3-VH*	20.9	Varuna
				*Oil-B7-2-DE*	19.8	Donskaja-IV
				*Oil-B7-3-DE*	19.5	Donskaja-IV
				*Oil-B7-4-DE*	23.0	Donskaja-IV

a *Identified as pleiotropic QTL by comparative QTL analysis between SE and ZE populations*.

b *Phenotypic variance (R^2^) explained by the QTL*.

The two *C-QTL*—*Oil-C-A8-1* and *Oil-C-B7-1* obtained from four populations, merged 11 and 12 component QTL, respectively. These two *C-QTL* collectively accommodated 19 of the 25 major component *Oil* QTL, and co-mapped with the two erucic acid QTL on LGs A08 and B07 with the trait enhancing alleles from the HE parents (Table 4, Supplementary Figure 3). The remaining QTL that did not merge into *C-QTL* were identified as “population-specific” QTL (Supplementary Table 3).

The allelic effects of the seven parents used for the development of the mapping populations were calculated for the nine *C-QTL*. Comparative allelic strength of each of the seven parents for the nine *C-QTL* was determined following Chardon et al. (2014). This analysis revealed that the parent J8, possessing highest oil content (~49%), had the most positive alleles at five of the nine *C-QTL*, followed by TM-4 and Varuna possessing most positive alleles at three and one *C-QTL*, respectively (Table 5).

**Table 5:** Estimation of allelic effects (in percent) of nine *Oil C-QTL* involving five SE mapping populations and comparative allelic status of the seven parental lines.

**Consensus QTL**	**Allelic effect of** ***Oil C-QTL***^**a**^					**Comparative allelic status of parents^b^**
	**EJ8^A8B7^**	**EPJ^A8B7^**	**VH ^A8B7^**	**DE^B7^**	**TD^A8^**	
*Oil-C-A7-1*	1.1 (J)	–	1.2 (H)	–	–	J > E = H = D = T = P > V
*Oil-C-A8-1*	3.0 (J)	3.0 (P)	2.0 (V)	–	1.5 (T)	J = P > V > T > D = E = H
*Oil-C-A10-1*	–	–	1.1 (V)	–	2.4 (T)	T > V > D = E = H = J = P
*Oil-C-B1-1*	1.0 (J)	0.9 (P)	–	–	–	J > P > E = H = D = T = V
*Oil-C-B2-1*	1.2 (E)	0.8 (E)	–	–	–	T = D = V = E = H > P > J
*Oil-C-B3-1*	0.7 (J)	1.8 (E)	–	–	–	J > E = H = D = T = V > P
*Oil-C-B4-1*	0.3 (J)	–	–	1.1 (D)	–	T = D > J > E = H = V = P
*Oil-C-B6-1*	1.3 (J)	–	–	1.0 (D)	–	J > D = T > E = H = V = P
*Oil-C-B7-1*	1.7 (J)	1.2 (P)	2.7 (V)	2.6 (D)	–	V > T = D > J > P > E = H

a *The parental lines contributing the trait enhancing allele are shown in parentheses*.

b *J, J8; E, EH-2; H, Heera; D, Donskaja-IV; T, TM-4; P, Pusa Jaikisan; V, Varuna*.

### QTL mapping of oil content in ZE populations

Of the three ZE populations, the QTL analysis of EJ8^Z^ population identified a total of 10 QTL from three environments distributed over five LGs (Supplementary Table 5). The phenotypic variance explained by the detected *Oil* QTL ranged from 4.5 to 21.1%. Of these, three were detected as major QTL with *R*^2^ greater than 10.0%. Eight of the 10 QTL had the positive allele from J8, while EH-2 contributed positive alleles to the remaining two QTL.

The DE^Z^ population detected a total of seven QTL from three year data. These QTL were distributed over four LGs (Supplementary Table 5). All the seven QTL were detected as major effect QTL with *R*^2^ varying from 10.8 to 24.2%. Six of the seven QTL had the trait enhancing allele from the high oil parent, Donskaja-IV.

The QTL re-analysis in VH^Z^ population (Jagannath et al., 2011) with phenotyping data of one location identified a total of two QTL on LGs A02 and A03, of which the QTL on LG A02 was detected as a major QTL. Both the QTL had the trait enhancing allele from the low oil content parent Heera (Supplementary Table 5).

*Oil* QTL analysis from the three ZE populations detected a total of 19 QTL including 10 QTL from EJ8^Z^ and seven QTL from DE^Z^ populations which were phenotyped in three environments, and two QTL from VH^Z^ population that was phenotyped only in one environment. However, in all the three ZE populations, no *Oil* QTL was detected in the erucic acid QTL regions of LGs A08 and B07 unlike the five SE populations that had most of the major effect QTL influencing oil content in that region. Of the 19 QTL detected in ZE populations, 11 were detected as major QTL with *R*^2^ more than 10.0%. Due to lack of sufficient number of component QTL from more than one mapping population in each of the 18 linkage groups, QTL meta-analysis of oil content in ZE populations was not undertaken.

### Identification of novel and common QTL between SE and ZE populations

**Figure 1:**
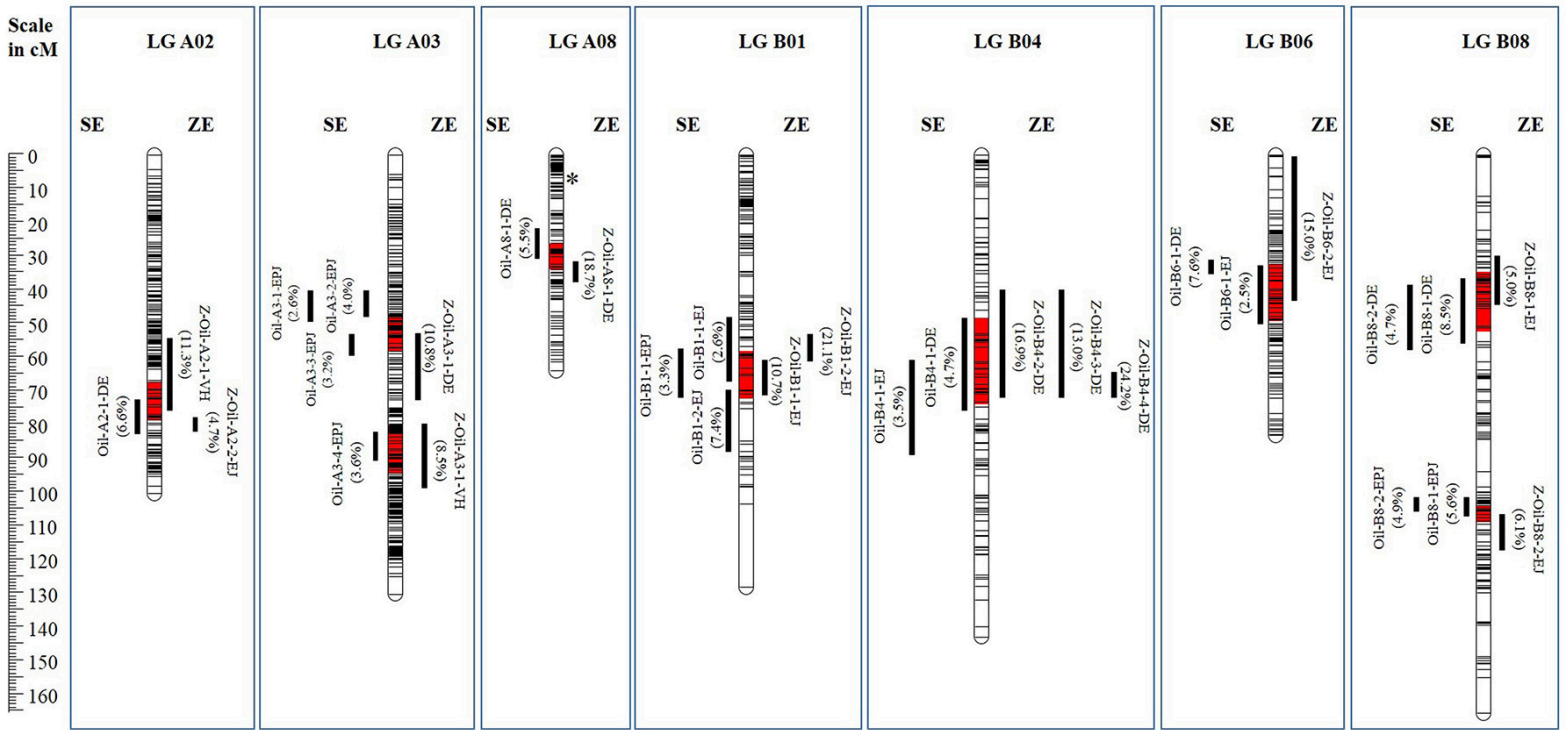
“Common” *Oil* QTL between SE and ZE populations identified by QTL meta-analysis. Meta-QTL regions are highlighted in red. Values in parentheses represent the phenotypic variance explained (*R*^2^) of the respective component QTL. Asterisk on LG A08 marks the map position of the *FAE1.1* gene.

QTL meta-analysis between the *Oil* QTL of SE populations and ZE populations was performed to identify the “common” QTL between the two types of populations. The analysis revealed the merger of 17 SE *Oil* QTL with 13 ZE *Oil* QTL and resulted in identification of nine “common” *Oil* QTL between SE and ZE populations distributed over seven LGs (A02, A03, A08, B01, B04, B06, and B08) (Figure 1). The remaining six QTL discerned in the ZE populations that did not merge to form “common” QTL, were identified as “novel” QTL—specific to ZE populations (Table 6). Of these six “novel” QTL, J8 had the trait enhancing alleles at four QTL, while Donskaja-IV and EH-2 contributed the positive alleles at one “novel” QTL, each.

**Table 6:** Details of the “novel” oil QTL identified in the three ZE populations.

**QTL name**	**LG**	**Position (cM)**	**LOD score**	**Additive effect**	***R*^2^ (%)^a^**	**Interval (cM)**	**Source of trait enhancing allele**
*Z-Oil-A2-1-EJ*	A02	1.1	4.6	0.5	7.5	0.0–2.6	J8
*Z-Oil-A5-1-EJ*	A05	49.5	3.3	0.5	7.2	23.8–56.1	J8
*Z-Oil-A5-1-DE*	A05	93.6	4.4	1.4	20.4	84.8–101	Donskaja-IV
*Z-Oil-B4-1-DE*	B04	0.0	2.8	−0.9	12.1	0.0–2.1	EH-2
*Z-Oil-B6-1-EJ*	B06	10.7	3.3	0.4	5.4	4.8–20.0	J8
*Z-Oil-B6-3-EJ*	B06	79.6	2.7	0.4	4.5	75.6–80.6	J8

a *Phenotypic variance (R^2^) explained by the QTL*.

**Figure 2:**
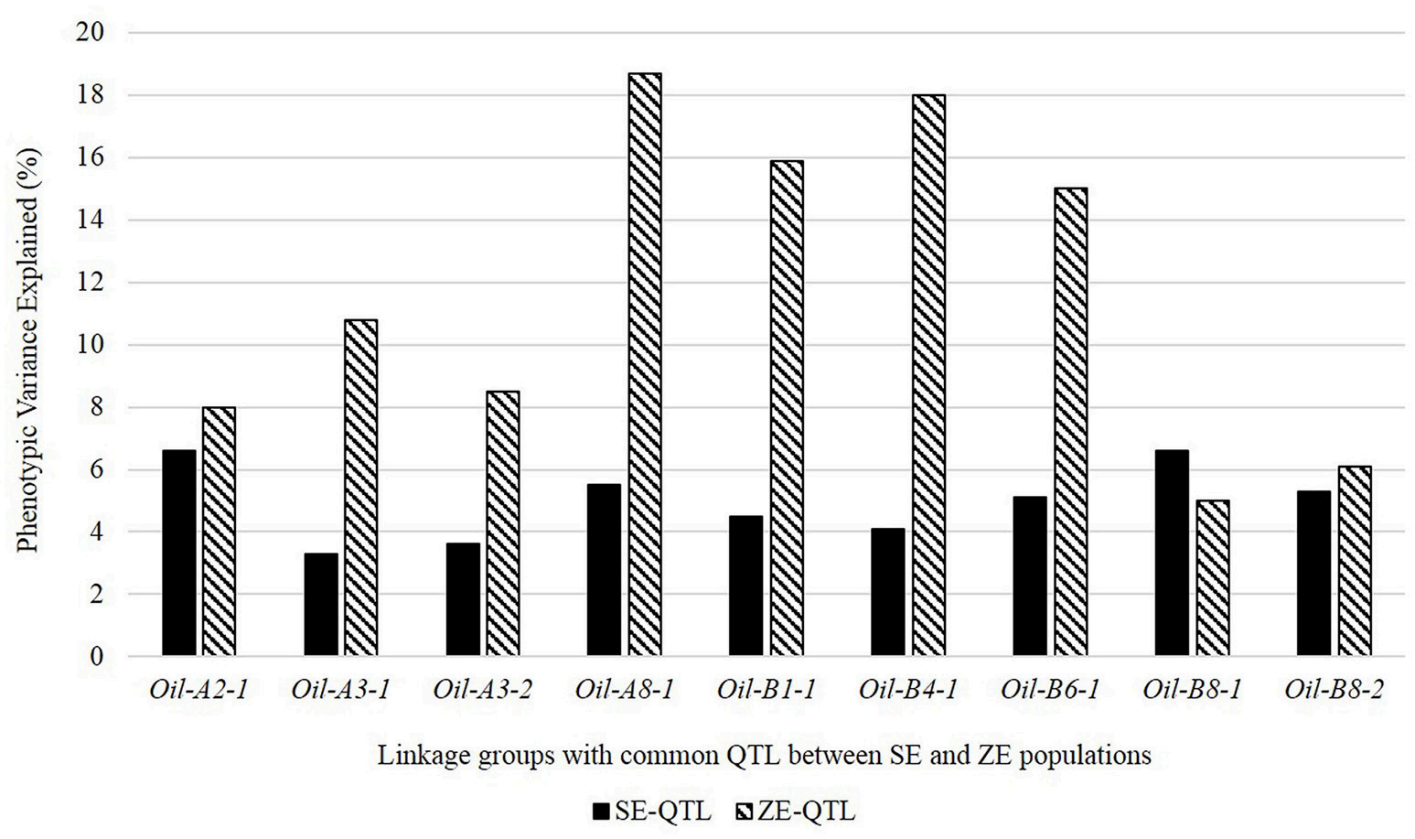
Comparison of average phenotypic variance explained (*R*^2^) by “common” QTL identified between SE and ZE populations after QTL meta-analysis.

The allelic strength of the five parental lines at four of the nine “common” QTL merging the QTL from the same source populations (LG A08 in DE, B01 in EJ8, B04 in DE, and B06 in EJ8) identified— J8 and Donskaja-IV possessing the positive alleles for two “common” QTL, each. The allelic effects of these parental lines at corresponding “common” QTL were observed to have increased in ZE populations as compared to the SE populations (Table 7). At eight of the nine “common” QTL, a comparison of average *R*^2^ values of these nine “common” QTL between SE and ZE populations showed a general trend of increase in R^2^ values of ZE *Oil* QTL as compared to the SE *Oil* QTL (Figure 2). Identification of the “novel” QTL, and increased allelic effects and *R*^2^ values of SE QTL at “common” QTL therefore, revealed a significant change in the landscape of oil QTL in ZE background.

**Table 7:** Comparison of allelic effects (%) of *Oil* QTL underlying the “common” QTL between SE and ZE background in the mapping populations resourced from the same parental crosses.

**LG**	**Component QTL**		**Allelic effect of component QTL**^**a**^			
			**SE populations**		**ZE populations**	
	**SE population**	**ZE population**	**EJ8^A8B7^**	**DE^B7^**	**EJ8^Z^**	**DE^Z^**
A08	*Oil-A8-1-DE*	*Z-Oil-A8-1-DE*	–	1.3 (D)	–	2.2 (D)
B01	*Oil-B1-1-EJ, Oil-B1-2-EJ*	*Z-Oil-B1-1-EJ, Z-Oil-B1-2-EJ*	1.0 (J)	–	1.2 (J)	–
B04	*Oil-B4-1-DE*	*Z-Oil-B4-2-DE, Z-Oil-B4-3-DE, Z-Oil-B4-4-DE*	–	1.1 (D)	–	2.7 (D)
B06	*Oil-B6-1-EJ*	*Z-Oil-B6-2-EJ*	1.3 (J)	–	1.4 (J)	–

a *The parental lines contributing the trait enhancing allele are shown in parentheses; J, J8; E, EH-2; H, Heera; D, Donskaja-IV; V, Varuna*.

The 15 multi-population QTL identified in the present study (Table 8) were projected on to the reference genome of *B. juncea* (Yang et al., 2016), *B. rapa* (Wang et al., 2011), *B. nigra* (Yang et al., 2016), and *B. napus* (Chalhoub et al., 2014), using the sequences of the PCR primers flanking the identified QTL through “BLASTn” tool available in the BRAD database (http://brassicadb.org/brad/blastPage.php). Mapping of the QTL detected on A-subgenome of *B. juncea* identified 17 corresponding QTL (Liu et al., 2016; Raboanatahiry et al., 2017) distributed over three A-subgenome LGs of *B. napus* genome. In the absence of any QTL data from *B. nigra* and B-subgenome of *B. carinata* for oil content, no such comparative analysis could be undertaken for the B-subgenome *Oil* QTL of *B. juncea*.

**Table 8:** Summary of *Oil* QTL identified in *B. juncea* and their physical positions on the reference genomes of *B. rapa, B. nigra, B. juncea*, and *B. napus*.

**Map positions of** ***Oil*** **QTL identified in** ***B. juncea***			**Physical positions on reference genomes**				**Corresponding** ***Oil*** **QTL identified on the A sub-genome of B. *napus*^*f*^**	
**Name of the QTL**	**LG**	**CI on INT Map (cM)**	***B. rapa* (Mb)^c^**	***B. nigra* (Mb)^d^**	***B. juncea* (Mb)^d^**	***B. napus* (Mb)^e^**	**Name of the QTL**	**Physical positions (Mb)**
*Oil-A2-1*^*a*^	A02	67.4–78.5	21.65–23.10	–	31.44–32.34	20.72–21.85	–	–
*Oil-A3-1*^*a*^	A03	47.3–57.9	11.06–13.44	–	13.75–16.39	10.16–12.54	qOC-A3-5-KN	8.99–14.92
*Oil-A3-2*^*a*^	A03	80.4–94.2	16.86–20.57	–	21.13–26.96	16.00–19.38	qOC-A3-DY, qOC-A3-RNSL, qOC-A3-4-TN	15.09–20.77
*Oil-C-A7-1*^*b*^	A07	45.9–70.1	23.36–23.39	–	32.17–32.22	21.84–21.87	–	–
*Oil-C-A8-1*^*b*^	A08	7.8–10.2	10.07–12.91	–	13.63–17.57	8.76–11.16	qOC-A8-2-KN, qOC-A8-3-KN, qOC-A8-4-KN, qOC-A8-5-KN, qOC-A8-6-KN, qOC-A8-7-KN, qOC-A8-8-KN, qOC-A8-TN, qOC-A8-RNSL	8.03–15.4
*Oil-A8-1*^*a*^	A08	26.3–33.8	16.05–17.78		20.81–22.72	14.30–15.98	–	–
*Oil-C-A10-1*^*b*^	A10	6.3–25.2	1.77–11.92	–	4.51–14.58	1.58–13.15	qOC-A10-DY, qOC-A10-2-KN, qOC-A10-TN, qOC-A10-3-KN	0.82–14.08
*Oil-B1-1*^*a*^ *Oil-C-B1-1*^*b*^	B01	56.6–73.3	–	2.89–6.52	3.88–9.08	–	–	–
*Oil-C-B2-1*^*b*^	B02	66.0–83.0	–	34.34–42.15	3.24–14.48	–	–	–
*Oil-C-B3-1*^*b*^	B03	47.5–73.8	–	12.93–30.21	23.70–32.41	–	–	–
*Oil-B4-1*^*a*^ *Oil-C-B4-1*^*b*^	B04	46.0–74.7	–	7.70–12.65	7.59–10.86	–	–	–
*Oil-B6-1*^*a*^ *Oil-C-B6-1*^*b*^	B06	32.4–50.1		16.21–20.37	15.64–19.45	–	–	–
*Oil-C-B7-1*^*b*^	B07	50.8–59.3	-	32.53–34.11	8.35–10.18	–	–	–
*Oil-B8-1*^*a*^	B08	34.7–52.2		5.76–25.64	7.38–24.59	–	–	–
*Oil-B8-2*^*a*^	B08	104.0–108.7		39.33–40.58	58.16–60.64	–	–	–

a *Common QTL between SE and ZE populations*.

b *C-QTL identified from five SE populations*.

c Wang et al., 2011;

d Yang et al., 2016;

e Chalhoub et al., 2014;

f *Liu et al., 2016 and Raboanatahiry et al., 2017*.

### Pleiotropic effect of erucic acid on oil content

**Figure 3:**
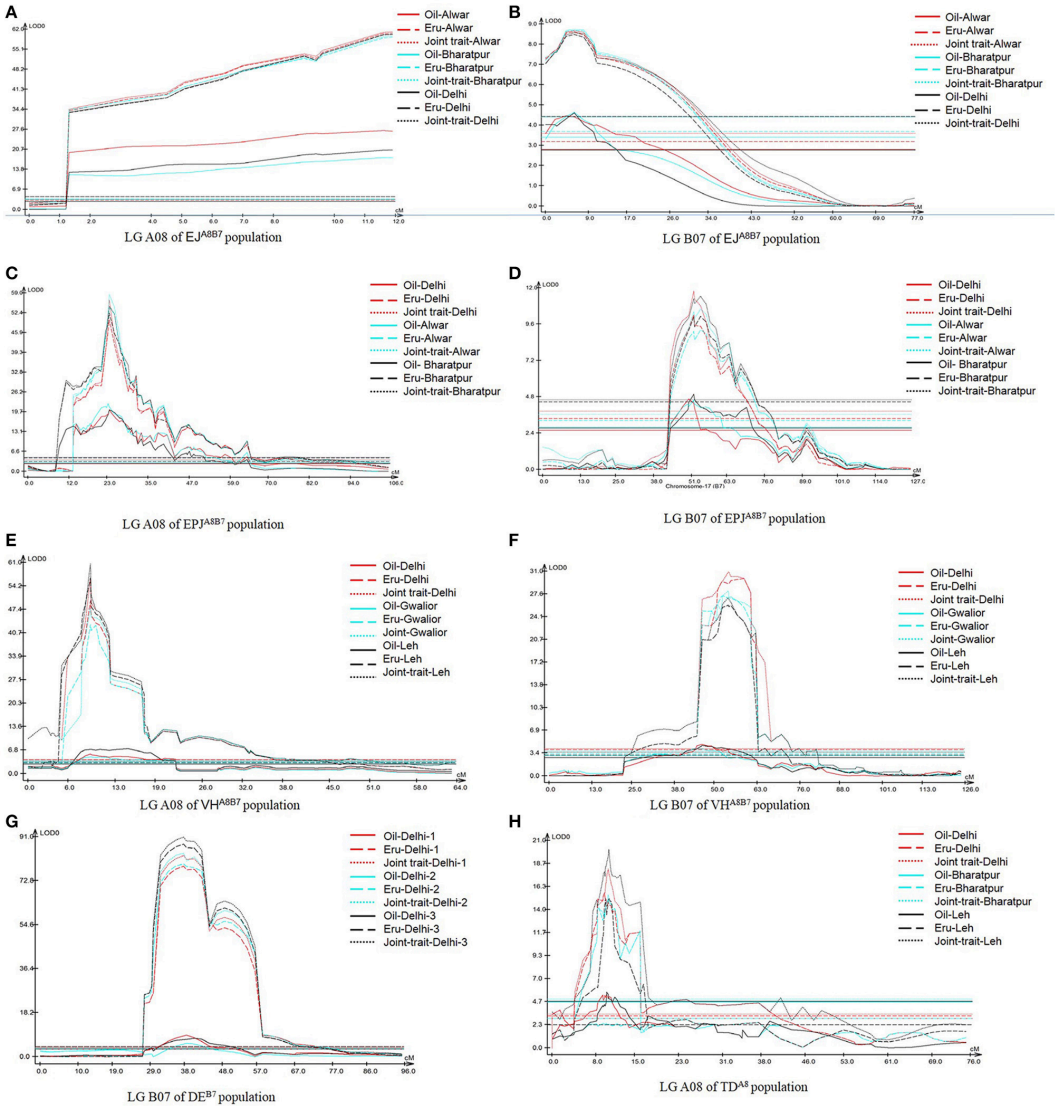
Multi-trait mapping (Mt-CIM) of seed oil content and erucic acid content in five SE bi-parental mapping populations of *B. juncea* showing pleiotropism between erucic acid and oil content (**A**: LG A08 of EJ8^A8B7^ population, **B**: LG B07 of EJ8^A8B7^ population, **C**: LG A08 of EPJ^A8B7^ population, **D**: LG B07 of EPJ^A8B7^ population, **E**: LG A08 of VH^A8B7^ population, **F**: LG B07 of VH^A8B7^ population, **G**: LG B07 of DE^B7^ population, and **H**: LG A08 of TD^A8^ population). The horizontal lines indicate LOD significance threshold.

As the investigated traits—erucic acid and oil content were positively correlated (Supplementary Table 1) and were identified as major QTL for both the traits in overlapping intervals on LGs A08 and B07, Mt-CIM (Jiang and Zeng, 1995) was used to distinguish whether the correlation was an artifact due to two closely-linked QTL or a single QTL was affecting both the traits through pleiotropy. The analysis was undertaken for the five SE populations (EJ8^A8B7^, EPJ^A8B7^, VH^A8B7^, DE^B7^, and TD^A8^) for LGs A08 and B07. In all the five SE populations, a joint analysis on LGs A08 and B07, revealed a similar pattern of LOD profile for the joint trait and for the two individual traits. These QTL were significant for both the individual as well as joint traits in all the environments (Figure 3), indicating existence of strong pleiotropy between the two traits. Hence the two *C-QTL, Oil-C-A8-1* on LG A08 and *Oil-C-B7-1* on LG B07 in SE populations, which were not detected in the corresponding ZE populations, were designated as pleiotropic QTL. The effect of pleiotropy was also evident from a significant change in QTL landscape in ZE background (Figure 2, Table 6).

## Discussion

Breeding for higher seed oil content in both HE and ZE mustard is critical for enhancing the productivity of the mustard crop. To address this problem effectively, seven inbred parental lines containing significant phenotypic variation for the trait (ranging from 37% to 49%) in the *B. juncea* germplasm were used for the development of two sets of bi-parental mapping populations—one set of bi-parental mapping populations segregating for erucic acid content (SE populations) and the other set consisting of only zero-erucic DH lines (ZE populations). A comparative QTL analysis between the two types of mapping populations has provided a detailed insight into the QTL landscape for seed oil content trait in *B. juncea*.

Seven inbred parental lines from which five SE and three ZE mapping populations were sourced consisted of lines with very high (49%), intermediate (41 to 44%), and low (37%) oil content-almost representing the maximum phenotypic variation reported for the trait in the *B. juncea* germplasm. The SE mapping populations showed higher mean values and presence of transgressive segregation in both the directions as compared to the ZE populations for oil content in the study. This difference in the mean and the spectrum of transgressive segregation observed between the two types of populations could probably be due to the influence of erucic acid on oil content as these two traits are positively correlated (Supplementary Table 1). Gu et al. (2017) reported positive correlation (*r* = 0.483) between oil content and long chain fatty acid particularly erucic acid in a set of lines of *B. napus* consisting of ultrahigh (>55%), high (50–55%), medium (40–50%), and low (30–40%) oil groups.

QTL analysis in five SE populations revealed distribution of *Oil* QTL throughout the genome (in 16 of the 18 LGs) with a sizable number of minor QTL having *R*^2^ less than 10%. Our results in *B. juncea*, support the earlier findings in rapeseed that the *Oil* QTL are distributed throughout the genome (Wang et al., 2013; Jiang et al., 2014). Comparison of *Oil* QTL data between SE and ZE populations revealed that none of the 23 QTL identified in SE populations on LGs A08 and B07 that co-mapped to the erucic acid QTL regions were detected in ZE populations. These *Oil* loci constitute 76% of the major QTL detected in the five SE populations (19 of the 25 major QTL) and merge into two consensus QTL in SE populations (*Oil-C-A8-1* on LG A08 and *Oil-C-B7-1* on LG B07). The multi-trait analysis for erucic acid and oil content on these two LGs also indicated existence of a strong pleiotropic effect between these two traits. Hence, these two *C-QTL* were designated as “Pleiotropic” QTL. It indicates that these two consensus *Oil* QTL, although the key QTL in SE populations, cannot be used to improve the oil content in ZE *B. juncea*.

Pleiotropic effect of erucic acid on oil content has also been reported earlier in both *B. napus* (Ecke et al., 1995; Burns et al., 2003) and *B. juncea* (Jagannath et al., 2011). Ecke et al. (1995) interpreted this occurrence owing to increase in the molecular mass of the carbon atoms of fatty acids during the elongation of oleic acid to erucic acid and resulting in a corresponding increase in the oil content in *B. napus*. As erucic acid is the major fatty acid (≥45% of the fatty acid pool) in *B. juncea* as in case of non-Canola *B. napus* and shows high positive correlation with oil content, similar mechanisms should be operational in *B. juncea* as well. A supporting evidence to this fact was provided by Katavic et al. (2000) by over-expression of *FAE1* (fatty acid elongase) gene in transgenic *B. napus* that showed an increase in erucic acid content up to 16.8% and a simultaneous increase in the oil content by 10.9% in non-Canola *B. napus* cv. Hero. The pleiotropic *Oil* QTL on the LG A08 (*Oil-C-A8-1*) identified in the present study in *B. juncea* also corresponds to the physical position in *B. napus* where maximum number of *Oil* QTL from multiple populations have been mapped (Table 8) (Liu et al., 2016; Raboanatahiry et al., 2017) indicating that these QTL in *B. napus* could also be “pleiotropic” QTL. However, Delourme et al. (2006) while comparing the QTL data of oil content from two bi-parental populations of *B. napus* interpreted that the *Oil* QTL detected in the erucic acid region of LG A08 could be due to linkage and not due to pleiotropy. This contention was based on the fact that a population (DY population) segregating for erucic acid detected no *Oil* QTL in the erucic acid region of LG A08 while another population (RNSL population) that was not segregating for erucic acid detected an *Oil* QTL in the corresponding region. Our results in the present study also detected an *Oil* QTL (*Oil-A8-1*) in the LG A08 in both SE and ZE populations of DE which does not segregate for the A08 erucic acid locus. The physical position of this *Oil* QTL is 5.98 Mb away from the pleiotropic QTL locus *Oil-C-A8-1* (Table 8).

In addition to the two pleiotropic QTL (*Oil-C-A8-1* and *Oil-C-B7-1*), this study has identified seven *C-QTL* (Table 5) and two major effect “population-specific” QTL–*Oil-A3-1-TD* and *Oil-B3-1-TD* (Supplementary Table 3) with significant contribution to the trait variability. The comparative allelic effects deciphered at the seven *C-QTL* identified J8 as the most desirable parental line for four loci (*Oil-C-A7-1, Oil-C-B1-1, Oil-C-B3-1*, and *Oil-C-B6-1*) and TM-4 for the remaining three loci (*Oil-C-A10-1, Oil-C-B2-1*, and *Oil-C-B4-1*)—these loci can be deployed in marker assisted breeding for improvement of oil content in HE mustard.

In the present study, we observed significant changes in the genetic landscape of *Oil* QTL when the influence of the erucic acid locus was absent. Eight out of the nine common *Oil* QTL between SE and ZE populations showed an upward trend in the phenotypic variance explained and also increase in the allelic effects of the respective parent in the ZE populations. It could probably be due to sharing of that part of the phenotypic variance owing to pleiotropic effect in SE populations that is reallocated among the *Oil* QTL in ZE populations when the pleiotropic effect of erucic acid is absent. Due to this reason, five QTL on LGs A03 (*Oil-A3-1*), A08 (*Oil-A8-1*), B01 (*Oil-B1-1*), B04 (*Oil-B4-1*), and B06 (*Oil-B6-1*) which were detected as minor QTL in SE populations were detected as major QTL in ZE populations (Figure 2). In addition, the appearance of six “novel” QTL in ZE populations (Table 6) could probably be due to exclusion of the effect of erucic acid on seed oil content. Jagannath et al. (2011) also reported a similar observation in *B. juncea* while comparing the SE and ZE QTL information from two mapping populations that were derived from one bi-parental cross. The present study, therefore, suggests that the genetic analysis of oil content in populations with high erucic acid content may not be relevant in breeding for high oil content in ZE mustard.

## Conclusion

The present study incorporated multiple mapping populations derived from seven parental lines with significant variation for oil content, including the pureline J8 with an oil content of ~49%. At four of the “common” QTL and four of the “novel” QTL identified in this study, J8 holds stronger alleles as compared to the other parental lines, positioning it as an excellent source for improvement of oil content in ZE background. The nine “common” QTL (Figure 1) and the six “novel” QTL (Table 6) identified here can be highly useful in compensating for the reduction in oil content in “0” erucic lines. Fine mapping of the important loci identified here will allow identification of the candidate genes and their interactions involved in oil content variation in *B. juncea*.

## Author contributions

KR and BY contributed equally and carried out the phenotyping and mapping work. SY helped in field experiments and data analysis. VG helped with genotyping. AM developed microspore derived F_1_DH mapping populations. DP and AP conceived and supervised the overall study and along with KR wrote the manuscript. All authors read and approved the final manuscript.

### Conflict of interest statement

The authors declare that the research was conducted in the absence of any commercial or financial relationships that could be construed as a potential conflict of interest.
