# Supplementary material for: QTL Landscape for Oil Content in Brassica juncea: Analysis in Multiple Bi-Parental Populations in High and “0” Erucic Background
Source: Front Plant Sci. 2018 Oct 16;9:1448. doi: 10.3389/fpls.2018.01448 (PMC6198181; doi:10.3389/fpls.2018.01448)
Supplement: Supplementary file 1 [file Table_1.DOCX]

**Supplementary Table 1**. Correlation coefficients between erucic acid content and seed oil content in five SE bi-parental mapping populations

| Environment | EJ8 ^A8B7^ | EPJ ^A8B7^ | VH ^A8B7^ | DE ^B7^ | TD ^A8^ |
| --- | --- | --- | --- | --- | --- |
| Environment 1 | 0.743** | 0.735** | 0.546** | 0.543** | 0.645** |
| Environment 2 | 0.770** | 0.720** | 0.643** | 0.390** | 0.585** |
| Environment 3 | 0.694** | 0.663** | 0.669** | 0.426** | 0.606** |

**Significant at 1%
