# Supplementary material for: QTL Landscape for Oil Content in Brassica juncea: Analysis in Multiple Bi-Parental Populations in High and “0” Erucic Background
Source: Front Plant Sci. 2018 Oct 16;9:1448. doi: 10.3389/fpls.2018.01448 (PMC6198181; doi:10.3389/fpls.2018.01448)
Supplement: Supplementary file 2 [file Table_2.DOCX]

**Supplementary Table 2**. Results of QTL analysis of erucic acid content (*Eru*) in five SE mapping populations

| **QTL name** | **LG** | **Position** | **LOD score** | **Additive effect** | **PVE (%)** | **Interval (cM)** | **Environment** | **Source of trait enhancing allele** | **Position of *FAE1* gene (cM)** |
| --- | --- | --- | --- | --- | --- | --- | --- | --- | --- |
| EJ8^A8B7^ Population | | | |  |  |  |  |  |  |
| *Eru-A5-1-EJ* | A05 | 5.5 | 2.9 | -1.7 | 1.2 | 3.2 - 7.5 | Bharatpur | EH-2 |  |
| *Eru-A61-EJ* | A06 | 20.2 | 2.6 | -1.5 | 1.0 | 10.7 - 26.3 | Bharatpur | EH-2 |  |
| *Eru-A8-1-EJ* | A08 | 11.6 | 63.8 | 12.5 | 64.8 | 10.6 - 11.6 | Alwar | J8 | 11.9 |
| *Eru-A8-2-EJ* | A08 | 11.6 | 61.3 | 12.4 | 63.7 | 10.6 - 11.6 | Bharatpur | J8 |  |
| *Eru-A8-3-EJ* | A08 | 11.6 | 62.3 | 12.4 | 64.4 | 10.6 - 11.6 | Delhi | J8 |  |
| *Eru-B7-1-EJ* | B07 | 7.0 | 43.4 | 8.4 | 28.7 | 4.9 - 8.3 | Alwar | J8 | 6.5 |
| *Eru-B7-2-EJ* | B07 | 7.0 | 43.4 | 8.5 | 29.7 | 5.9 - 8.4 | Bharatpur | J8 |  |
| *Eru-B7-3-EJ* | B07 | 7.0 | 41.2 | 8.2 | 27.5 | 5.7 - 8.6 | Delhi | J8 |  |
| EPJ^A8B7^ Population | | |  |  |  |  |  |  |  |
| *Eru-A4-1-EPJ* | A04 | 12.4 | 2.6 | 1.4 | 1.1 | 10.1 - 18.7 | Delhi | Pusa Jaikisan |  |
| *Eru-A8-1-EPJ* | A08 | 23.7 | 50.9 | 11.7 | 55.5 | 14.0 - 31.7 | Delhi | Pusa Jaikisan | 23.7 |
| *Eru-A8-2-EPJ* | A08 | 23.7 | 55.6 | 12.4 | 59.5 | 23.4 - 34.7 | Alwar | Pusa Jaikisan |  |
| *Eru-A8-3-EPJ* | A08 | 23.7 | 56.7 | 11.2 | 60.2 | 23.4 - 34.7 | Bharatpur | Pusa Jaikisan |  |
| *Eru-B2-1-EPJ* | B02 | 147.4 | 2.8 | -2.0 | 1.3 | 140.5 - 149.4 | Delhi | EH-2 |  |
| *Eru-B5-1-EPJ* | B05 | 0.0 | 5.0 | -2.7 | 2.1 | 0 - 2.9 | Delhi | EH-2 |  |
| *Eru-B7-1-EPJ* | B07 | 53.0 | 39.5 | 8.4 | 29.0 | 52.6 - 55.1 | Delhi | Pusa Jaikisan | 51.7 |
| *Eru-B7-2-EPJ* | B07 | 54.0 | 37.1 | 8.4 | 28.5 | 52.9 - 56.0 | Alwar | Pusa Jaikisan |  |
| *Eru-B7-3-EPJ* | B07 | 54.0 | 38.4 | 7.8 | 29.9 | 52.7 - 55.8 | Bharatpur | Pusa Jaikisan |  |
| VH ^A8B7^Populationª | | |  |  |  |  |  |  |  |
| *Eru-A8-1-VH* | A08 | 9.3 | 59.8 | 12.4 | 61.3 | 9.0 - 10.7 | Delhi | Varuna | 9.3 |
| *Eru-A8-2-VH* | A08 | 9.3 | 53.2 | 11.5 | 56.3 | 8.5 - 10.7 | Gwalior | Varuna |  |
| *Eru-A8-1-VH* | A08 | 9.3 | 61.3 | 12.7 | 63.2 | 9.3 - 15.4 | Leh | Varuna |  |
| *Eru-B7-1-VH* | B07 | 54.2 | 43.4 | 8.1 | 38.4 | 51.3 - 54.7 | Delhi | Varuna | 54.2 |
| *Eru-B7-2-VH* | B07 | 54.2 | 41.4 | 7.7 | 33.2 | 50.7 - 61.2 | Leh | Varuna |  |
| *Eru-B7-3-VH* | B07 | 54.2 | 39.2 | 7.5 | 29.7 | 50.7 - 55.4 | Gwalior | Varuna |  |
| DE^B7^ Population | |  |  |  |  |  |  |  |  |
| *Eru-A3-1-DE* | A03 | 64.0 | 2.5 | -1.1 | 0.6 | 63.2 - 69.4 | Delhi Year 1 | EH-2 |  |
| *Eru-A5-1-DE* | A05 | 100.6 | 3.8 | -1.3 | 0.9 | 93.9 - 109.5 | Delhi Year 1 | EH-2 |  |
| *Eru-A7-1-DE* | A07 | 63.6 | 3.9 | -1.3 | 0.7 | 48.5 - 63.8 | Delhi Year 3 | EH-2 |  |
| *Eru-A8-1-DE* | A08 | 6.2 | 5.0 | -1.4 | 0.9 | 4.7 - 13.9 | Delhi Year 3 | EH-2 | 4.5^a^ |
| *Eru-A10-1-DE* | A10 | 11.2 | 5.5 | -1.7 | 1.1 | 7.7 - 12.3 | Delhi Year 3 | EH-2 |  |
| *Eru-B7-1-DE* | B07 | 37.7 | 88.2 | 13.5 | 92.2 | 36.1 - 38.3 | Delhi Year 1 | Donskaja-IV | 38.5 |
| *Eru-B7-2-DE* | B07 | 37.7 | 84.6 | 11.7 | 90.7 | 35.5 - 50.6 | Delhi Year 2 | Donskaja-IV |  |
| *Eru-B7-3-DE* | B07 | 37.7 | 97.4 | 14.0 | 89.1 | 35.9 - 38.2 | Delhi Year 3 | Donskaja-IV |  |
| TD^A8^Population | | |  |  |  |  |  |  |  |
| *Eru-A7-1-TD* | A07 | 45.8 | 2.5 | -2.6 | 4.9 | 38.6 - 47.5 | Bharatpur | Donskaja-IV |  |
| *Eru-A8-1-TD* | A08 | 10.2 | 17.3 | 6.8 | 51.1 | 9.0 - 10.7 | Delhi | TM-4 | 9.2 |
| *Eru-A8-2-TD* | A08 | 10.2 | 15.6 | 6.9 | 45.3 | 8.5 - 10.7 | Leh | TM-4 |  |
| *Eru-A8-3-TD* | A08 | 15.0 | 13.8 | 5.8 | 43.3 | 9.3 - 15.4 | Bharatpur | TM-4 |  |
| *Eru-B2-1-TD* | B02 | 106.3 | 2.6 | 2.4 | 5.7 | 99 - 115.1 | Leh | TM-4 |  |
| *Eru-B7-1-TD* | B07 | 0.0 | 4.5 | 2.0 | 3.7 | 0 - 1.9 | Delhi | TM-4 | 30.8^a^ |

^a^Tentatively placed through the common flanking markers as the *FAE1* gene marker did not show polymorphism
