# Supplementary material for: QTL Landscape for Oil Content in Brassica juncea: Analysis in Multiple Bi-Parental Populations in High and “0” Erucic Background
Source: Front Plant Sci. 2018 Oct 16;9:1448. doi: 10.3389/fpls.2018.01448 (PMC6198181; doi:10.3389/fpls.2018.01448)
Supplement: Supplementary file 3 [file Table_3.DOCX]

**Supplementary Table 3.** Results of QTL analysis of oil content (*Oil)* from five SE mapping populations

| **QTL name^a^** | **LG** | **Peak LOD Position (cM)** | **LOD score** | **Additive effect** | **PVE (%)** | **Interval (cM)** | **Environment** | **Source of trait enhancing allele** | **Mapping position of two *FAE1* genes (cM)** |
| --- | --- | --- | --- | --- | --- | --- | --- | --- | --- |
| **EJ8^A8B7^ Population** | | | | | | | | | |
| *Oil-A7-1-EJ* | A07 | 58.3 | 3.1 | 0.5 | 3.3 | 56.4 - 63.6 | Alwar | J8 |  |
| *Oil-A7-2-EJ* | A07 | 67.9 | 5.5 | 0.7 | 6.2 | 63.5 - 71.9 | Delhi | J8 |  |
| *Oil-A8-1-EJ* | A08 | 9.4 | 19.8 | 1.5 | 28.2 | 8 - 9.6 | Delhi | J8 | 11.9 |
| *Oil-A8-2-EJ* | A08 | 11.6 | 17.9 | 1.3 | 30.5 | 1.2 - 11.9 | Bharatpur | J8 |  |
| *Oil-A8-3-EJ* | A08 | 11.6 | 28.0 | 1.8 | 43.3 | 10.5 - 11.9 | Alwar | J8 |  |
| *Oil-B1-1-EJ* | B01 | 42.9 | 2.5 | 0.4 | 2.6 | 30.1 - 53.4 | Alwar | J8 |  |
| *Oil-B1-2-EJ* | B01 | 56.6 | 6.7 | 0.8 | 7.4 | 53.9 - 62 | Delhi | J8 |  |
| *Oil-B2-1-EJ* | B02 | 21.5 | 4.6 | -0.6 | 5.0 | 17.3 - 31.1 | Alwar | EH-2 |  |
| *Oil-B2-2-EJ* | B02 | 24.3 | 6.9 | -0.8 | 7.7 | 22.3 - 29.1 | Delhi | EH-2 |  |
| *Oil-B3-1-EJ* | B03 | 36.0 | 2.6 | 0.5 | 3.8 | 34.8 - 38.6 | Bharatpur | J8 |  |
| *Oil-B4-1-EJ* | B04 | 47.3 | 3.1 | 0.5 | 3.5 | 42.1 - 56.6 | Alwar | J8 |  |
| *Oil-B6-1-EJ* | B06 | 46.3 | 2.6 | 0.5 | 2.5 | 37.7 - 55 | Delhi | J8 |  |
| *Oil-B7-1-EJ* | B07 | 5.3 | 10.3 | 1.0 | 12.2 | 0.0 - 9.4 | Delhi | J8 | 6.5 |
| *Oil-B7-2-EJ* | B07 | 6.0 | 11.4 | 1.0 | 13.7 | 3.1 - 9.8 | Alwar | J8 |  |
| *Oil-B7-3-EJ* | B07 | 7.0 | 8.5 | 0.9 | 12.8 | 2.9 - 16.8 | Bharatpur | J8 |  |
| **EPJ^A8B7^ Population** | | | | | | | | | |
| ***Oil-A1-1-EPJ*** | A01 | 59.3 | 3.0 | 0.4 | 3.1 | 56.8 - 61.1 | Delhi | Pusa Jaikisan |  |
| ***Oil-A3-1-EPJ*** | A03 | 37.5 | 2.5 | -0.4 | 2.6 | 36.9 - 37.7 | Delhi | EH-2 |  |
| ***Oil-A3-2-EPJ*** | A03 | 38.1 | 4.2 | -0.6 | 4.0 | 37.7 - 38.9 | Alwar | EH-2 |  |
| ***Oil-A3-3-EPJ*** | A03 | 47.1 | 2.8 | -0.5 | 3.2 | 43.3 - 48 | Bharatpur | EH-2 |  |
| ***Oil-A3-4-EPJ*** | A03 | 70.7 | 3.1 | -0.5 | 3.6 | 69.7 - 71.7 | Bharatpur | EH-2 |  |
| ***Oil-A4-1-EPJ*** | A04 | 0.0 | 4.2 | 0.7 | 4.5 | 0 - 1 | Alwar | Pusa Jaikisan |  |
| ***Oil-A4-2-EPJ*** | A04 | 0.0 | 3.5 | 0.5 | 4.0 | 0 - 1 | Delhi | Pusa Jaikisan |  |
| ***Oil-A4-3-EPJ*** | A04 | 12.4 | 3.5 | 0.6 | 4.4 | 9.5 - 23 | Bharatpur | Pusa Jaikisan |  |
| *Oil-A8-1-EPJ* | A08 | 22.4 | 18.8 | 1.5 | 30.1 | 11.7 - 23.7 | Bharatpur | Pusa Jaikisan | 23.7 |
| *Oil-A8-2-EPJ* | A08 | 23.4 | 24.4 | 1.9 | 34.3 | 13.2 - 31.6 | Alwar | Pusa Jaikisan |  |
| *Oil-A8-3-EPJ* | A08 | 23.7 | 23.2 | 1.5 | 35.2 | 13.2 - 34.7 | Delhi | Pusa Jaikisan |  |
| *Oil-B1-1-EPJ* | B01 | 60.8 | 2.7 | 0.5 | 3.3 | 53 - 63.1 | Bharatpur | Pusa Jaikisan |  |
| *Oil-B2-1-EPJ* | B02 | 88.5 | 4.5 | -0.6 | 6.5 | 87.5 - 90.4 | Delhi | EH-2 |  |
| *Oil-B2-2-EPJ* | B02 | 92.6 | 2.5 | -0.5 | 3.4 | 87.6 - 95.9 | Bharatpur | EH-2 |  |
| *Oil-B2-3-EPJ* | B02 | 124.4 | 2.6 | -0.5 | 3.8 | 123.4 - 127.6 | Bharatpur | EH-2 |  |
| *Oil-B3-1-EPJ* | B03 | 42.8 | 6.8 | -0.8 | 9.4 | 42 - 55.9 | Bharatpur | EH-2 |  |
| *Oil-B3-2-EPJ* | B03 | 44.4 | 8.2 | -1.0 | 9.8 | 41.3 - 56 | Alwar | EH-2 |  |
| *Oil-B3-3-EPJ* | B03 | 50.1 | 7.5 | -0.7 | 9.0 | 49.1 - 60.5 | Delhi | EH-2 |  |
| *Oil-B7-1-EPJ* | B07 | 56.0 | 7.2 | 0.7 | 9.2 | 53.2 - 59.2 | Delhi | Pusa Jaikisan | 51.7 |
| *Oil-B7-2-EPJ* | B07 | 56.0 | 7.3 | 0.9 | 8.4 | 53.0 - 68.4 | Alwar | Pusa Jaikisan |  |
| *Oil-B7-3-EPJ* | B07 | 57.0 | 4.6 | 0.7 | 6.1 | 52.0 - 67.7 | Bharatpur | Pusa Jaikisan |  |
| ***Oil-B8-1-EPJ*** | B08 | 103.1 | 5.1 | -0.7 | 5.6 | 102.3 - 104.1 | Alwar | EH-2 |  |
| ***Oil-B8-2-EPJ*** | B08 | 103.1 | 3.8 | -0.6 | 4.9 | 101.8 - 104.2 | Bharatpur | EH-2 |  |
| **VH^A8B7^ Population** | | | | | | | | | |
| *Oil-A7-1-VH* | A07 | 44.5 | 5.4 | -0.8 | 12.5 | 43.2 - 47.0 | Leh | Heera |  |
| *Oil-A7-2-VH* | A07 | 68.8 | 4.2 | -1.0 | 8.9 | 67.9 - 72.8 | Delhi | Heera |  |
| *Oil-A8-1-VH* | A08 | 10.1 | 5.6 | 1.0 | 15.2 | 8.3 - 10.7 | Gwalior | Varuna | 9.3 |
| *Oil-A8-2-VH* | A08 | 12.2 | 9.2 | 1.1 | 24.2 | 12.1 - 22.7 | Leh | Varuna |  |
| *Oil-A8-3-VH* | A08 | 15.9 | 6.0 | 1.3 | 13.8 | 12.1 - 17.7 | Delhi | Varuna |  |
| ***Oil-A9-1-VH*** | A09 | 21.6 | 2.7 | -0.7 | 6.8 | 19.7 - 26.3 | Gwalior | Heera |  |
| *Oil-A10-1-VH* | A10 | 20.6 | 5.4 | 1.2 | 11.3 | 19.9 - 38.8 | Delhi | Varuna |  |
| *Oil-A10-2-VH* | A10 | 23.2 | 2.9 | 0.8 | 7.3 | 22.7 - 30.2 | Gwalior | Varuna |  |
| *Oil-B7-1-VH* | B07 | 44.2 | 4.5 | 0.9 | 12.0 | 42.8 - 56.1 | Gwalior | Varuna | 54.2 |
| *Oil-B7-2-VH* | B07 | 46.3 | 4.5 | 1.0 | 9.4 | 40.6 – 61.0 | Delhi | Varuna |  |
| *Oil-B7-3-VH* | B07 | 60.0 | 8.1 | 1.1 | 20.9 | 55.4 - 62.4 | Leh | Varuna |  |
| ***Oil-B8-1-VH*** | B08 | 138.6 | 4.2 | -1.0 | 8.8 | 132.5 - 142.9 | Delhi | Heera |  |
| **DE^B7^ Population** | | | | | | | | | |
| ***Oil-A2-1-DE*** | A02 | 85.9 | 3.7 | -0.8 | 6.6 | 82.9 - 91 | Delhi Year 3 | EH-2 |  |
| ***Oil-A5-1-DE*** | A05 | 1.3 | 4.4 | -0.8 | 7.8 | 0 - 10.2 | Delhi Year 1 | EH-2 |  |
| ***Oil-A5-2-DE*** | A05 | 8.3 | 4.5 | -0.9 | 8.7 | 1.2 - 16.4 | Delhi Year 3 | EH-2 |  |
| ***Oil-A8-1-DE*** | A08 | 33.5 | 2.9 | 0.7 | 5.5 | 29.2 - 39.5 | Delhi Year 2 | Donskaja-IV | 4.5^b^ |
| ***Oil-A9-1-DE*** | A09 | 87.3 | 3.3 | -0.7 | 5.7 | 80.7 - 97 | Delhi Year 1 | EH-2 |  |
| *Oil-B4-1-DE* | B04 | 60.2 | 2.6 | 0.7 | 4.7 | 51.2 - 65.6 | Delhi Year 3 | Donskaja-IV |  |
| *Oil-B6-1-DE* | B06 | 45.3 | 4.1 | 0.8 | 7.6 | 44.6 - 47.1 | Delhi Year 2 | Donskaja-IV |  |
| ***Oil-B7-1-DE*** | B07 | 18.8 | 3.2 | -1.0 | 8.0 | 4.6 - 25.7 | Delhi Year 2 | EH-2 |  |
| *Oil-B7-2-DE* | B07 | 38.5 | 10.0 | 1.3 | 19.8 | 36.4 - 40.3 | Delhi Year 1 | Donskaja-IV | 38.5 |
| *Oil-B7-3-DE* | B07 | 38.5 | 9.6 | 1.5 | 19.5 | 36.2 - 41.4 | Delhi Year 2 | Donskaja-IV |  |
| *Oil-B7-4-DE* | B07 | 40.5 | 10.7 | 1.5 | 23.0 | 36.4 - 43.6 | Delhi Year 3 | Donskaja-IV |  |
| ***Oil-B8-1-DE*** | B08 | 38.0 | 4.5 | -0.8 | 8.5 | 37 - 46 | Delhi Year 2 | EH-2 |  |
| ***Oil-B8-2-DE*** | B08 | 38.0 | 2.6 | -0.7 | 4.7 | 37 - 50.8 | Delhi Year 3 | EH-2 |  |
| **TD^A8^ Population** | | | | | | | | | |
| ***Oil-A3-1-TD*** | A03 | 79.3 | 6.6 | -1.0 | 17.6 | 68.8 - 79.5 | Leh | Donskaja-IV |  |
| *Oil-A8-1-TD* | A08 | 9.9 | 4.8 | 0.9 | 12.4 | 5.2 - 14.0 | Leh | TM-4 | 9.2 |
| *Oil-A8-2-TD* | A08 | 10.0 | 5.2 | 1.1 | 17.0 | 0.0 - 10.8 | Delhi | TM-4 |  |
| *Oil-A10-1-TD* | A10 | 0.0 | 4.6 | 0.9 | 11.8 | 0.0 - 1.9 | Delhi | TM-4 |  |
| *Oil-A10-2-TD* | A10 | 11.1 | 7.3 | 1.6 | 22.4 | 9.5 - 13.3 | Bharatpur | TM-4 |  |
| ***Oil-B1-1-TD*** | B01 | 70.4 | 3.6 | 0.7 | 9.1 | 62.6 - 75.3 | Leh | TM-4 |  |
| ***Oil-B2-1-TD*** | B02 | 8.1 | 2.7 | 0.9 | 7.2 | 7.0 - 9.1 | Bharatpur | TM-4 |  |
| ***Oil-B3-1-TD*** | B03 | 88.8 | 4.3 | -1.1 | 11.6 | 86.1 - 90.8 | Bharatpur | Donskaja-IV |  |
| ***Oil-B7-1-TD*** | B07 | 1.9 | 3.3 | 0.8 | 8.1 | 0.6 - 5.1 | Delhi | TM-4 | 30.8^b^ |

^a^QTL shown in bold are identified as ‘population-specific’ QTL

^b^Tentatively placed through the common flanking markers as the *FAE1* gene marker did not show polymorphism
