# Supplementary material for: QTL Landscape for Oil Content in Brassica juncea: Analysis in Multiple Bi-Parental Populations in High and “0” Erucic Background
Source: Front Plant Sci. 2018 Oct 16;9:1448. doi: 10.3389/fpls.2018.01448 (PMC6198181; doi:10.3389/fpls.2018.01448)
Supplement: Supplementary file 5 [file Table_5.DOCX]

**Supplementary Table 5.** Results of QTL analysis of oil content (*Oil)* from three ZE populations

| **QTL name** | **LG** | **Position** | **LOD score** | **Additive effect** | **PVE (%)** | **Interval (cM)** | **Environment** | **Source of trait enhancing allele** |
| --- | --- | --- | --- | --- | --- | --- | --- | --- |
| **EJ8^Z^ Population** | | | | | | | | |
| *Z-Oil-A2-1-EJ* | A02 | 1.1 | 4.6 | 0.5 | 7.5 | 0.0 - 2.6 | Alwar | J8 |
| *Z-Oil-A2-2-EJ* | A02 | 72.6 | 2.7 | -0.4 | 4.7 | 71.7 - 73.3 | Delhi | EH-2 |
| *Z-Oil-A5-1-EJ* | A05 | 49.5 | 3.3 | 0.5 | 7.2 | 23.8 - 56.1 | Bharatpur | J8 |
| *Z-Oil-B1-1-EJ* | B01 | 49.2 | 5.7 | 0.5 | 10.7 | 46.6 - 68.1 | Delhi | J8 |
| *Z-Oil-B1-2-EJ* | B01 | 51.7 | 11.2 | 0.8 | 21.1 | 50.1 - 52.3 | Alwar | J8 |
| *Z-Oil-B6-1-EJ* | B06 | 10.7 | 3.3 | 0.4 | 5.4 | 4.8 - 20.0 | Alwar | J8 |
| *Z-Oil-B6-2-EJ* | B06 | 51.9 | 7.7 | 0.7 | 15.0 | 34.1 - 55.1 | Delhi | J8 |
| *Z-Oil-B6-3-EJ* | B06 | 79.6 | 2.7 | 0.4 | 4.5 | 75.6 - 80.6 | Delhi | J8 |
| *Z-Oil-B8-1-EJ* | B08 | 6.2 | 3.2 | 0.4 | 5.0 | 2.4 - 30.2 | Alwar | J8 |
| *Z-Oil-B8-2-EJ* | B08 | 90 | 3.5 | -0.4 | 6.1 | 85.5 - 91.8 | Delhi | EH-2 |
| **DE^Z^ Population** | | | | | | | | |
| *Z-Oil-A3-1-DE* | A03 | 60.0 | 2.9 | 1.2 | 10.8 | 58.1 - 62.2 | Delhi Year 2 | Donskaja-IV |
| *Z-Oil-A5-1-DE* | A05 | 93.6 | 4.4 | 1.4 | 20.4 | 84.8 – 101.0 | Delhi Year 2 | Donskaja-IV |
| *Z-Oil-A8-1-DE* | A08 | 38.6 | 4.2 | 1.4 | 18.7 | 37.0 - 42.9 | Delhi Year 2 | Donskaja-IV |
| *Z-Oil-B4-1-DE* | B04 | 0.0 | 2.8 | -0.9 | 12.1 | 0.0 - 2.1 | Delhi Year 1 | EH-2 |
| *Z-Oil-B4-2-DE* | B04 | 55.2 | 4.2 | 1.2 | 16.9 | 43.5 - 60.8 | Delhi Year 1 | Donskaja-IV |
| *Z-Oil-B4-3-DE* | B04 | 55.2 | 3.4 | 1.2 | 13.0 | 47.4 - 61.4 | Delhi Year 2 | Donskaja-IV |
| *Z-Oil-B4-4-DE* | B04 | 55.2 | 5.5 | 1.6 | 24.2 | 52.1 - 60.2 | Delhi Year 3 | Donskaja-IV |
| **VH^Z^ Population** | | | | | | | | |
| *Z-Oil-A2-1-VH* | A02 | 42.9 | 3.4 | -1.2 | 11.3 | 37.3 - 48.4 | Delhi | Heera |
| *Z-Oil-A3-1-VH* | A03 | 66.1 | 2.6 | -1.2 | 8.5 | 56.1 – 76.0 | Delhi | Heera |
