# Supplementary material for: QTL Landscape for Oil Content in Brassica juncea: Analysis in Multiple Bi-Parental Populations in High and “0” Erucic Background
Source: Front Plant Sci. 2018 Oct 16;9:1448. doi: 10.3389/fpls.2018.01448 (PMC6198181; doi:10.3389/fpls.2018.01448)
Supplement: Supplementary file 6 [file Presentation_1.PPTX]

## Slide 1
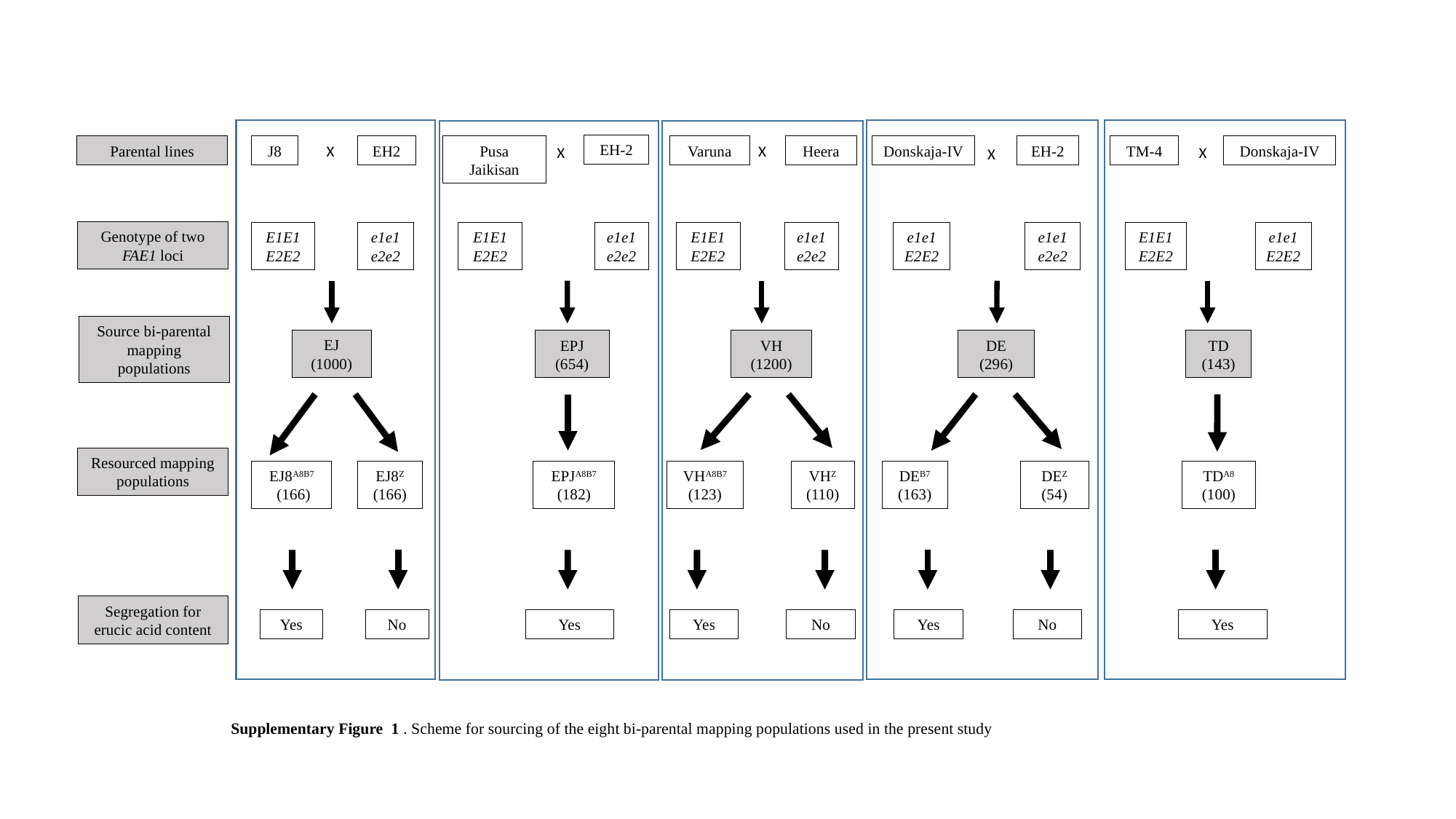

EH-2
EH-2
TM-4
Donskaja-IV
Donskaja-IV
Parental lines
J8
X
EH2
Varuna
Heera
Pusa Jaikisan
X
X
X
X
Genotype of two FAE1 loci
E1E1E2E2
e1e1e2e2
E1E1E2E2
e1e1e2e2
e1e1E2E2
e1e1e2e2
E1E1E2E2
e1e1E2E2
e1e1e2e2
E1E1E2E2
Source bi-parental mapping populations
EJ (1000)
EPJ (654)
VH (1200)
DE (296)
TD (143)
Resourced mapping populations
EJ8A8B7
 (166)
EJ8Z (166)
EPJA8B7 (182)
VHA8B7 (123)
VHZ (110)
DEB7 (163)
DEZ (54)
TDA8 (100)
Segregation for erucic acid content
Yes
No
Yes
Yes
No
Yes
No
Yes
Supplementary Figure 1 . Scheme for sourcing of the eight bi-parental mapping populations used in the present study
