# Supplementary material for: QTL Landscape for Oil Content in Brassica juncea: Analysis in Multiple Bi-Parental Populations in High and “0” Erucic Background
Source: Front Plant Sci. 2018 Oct 16;9:1448. doi: 10.3389/fpls.2018.01448 (PMC6198181; doi:10.3389/fpls.2018.01448)
Supplement: Supplementary file 7 [file Presentation_2.PPTX]

## Slide 1
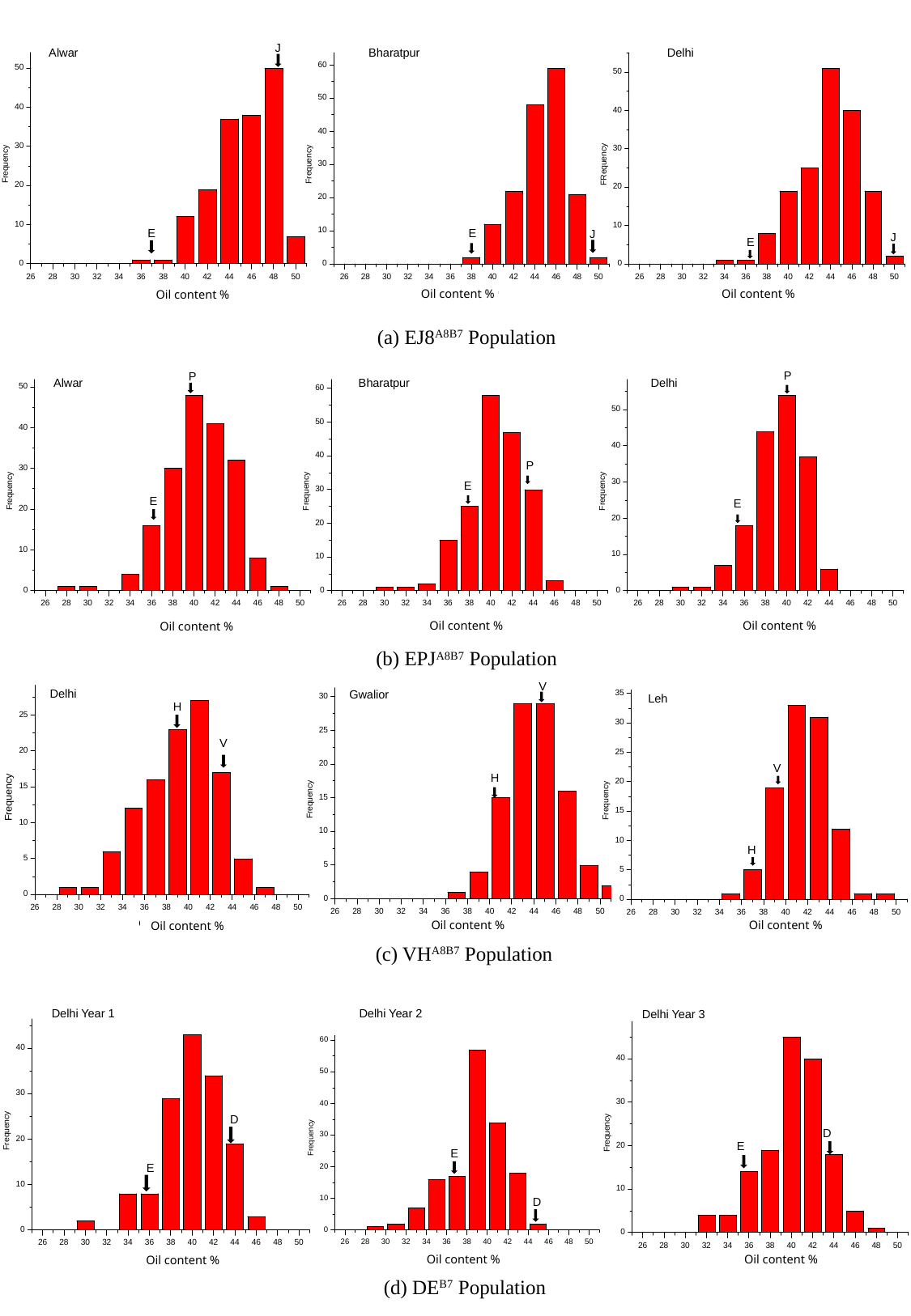

J
Alwar
E
Bharatpur
E
J
Delhi
J
E
(a) EJ8A8B7 Population
P
Alwar
E
Bharatpur
P
E
P
Delhi
E
(b) EPJA8B7 Population
H
Delhi
V
V
H
Gwalior
Leh
V
H
(c) VHA8B7 Population
Delhi Year 1
D
E
Delhi Year 3
D
E
Delhi Year 2
E
D
(d) DEB7 Population
Oil content %
Oil content %
Oil content %
Oil content %
Oil content %
Oil content %
Oil content %
Oil content %
Oil content %
Oil content %
Oil content %
Oil content %

## Slide 2
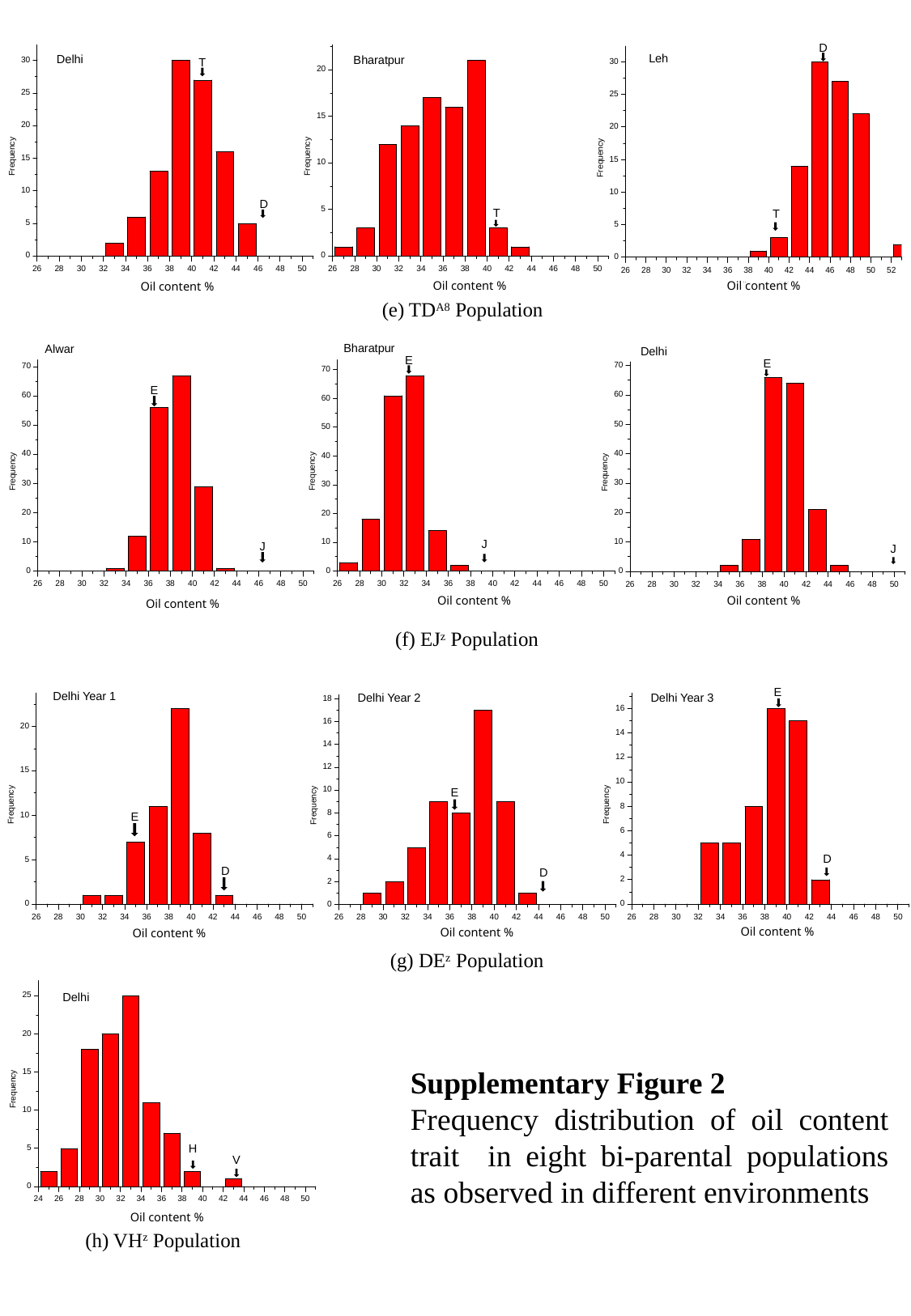

Bharatpur
T
Delhi
T
D
D
Leh
T
(e) TDA8 Population
Bharatpur
E
J
Alwar
E
J
Delhi
E
J
(f) EJz Population
Delhi Year 1
E
D
Delhi Year 2
E
D
E
Delhi Year 3
D
(g) DEz Population
Delhi
H
V
(h) VHz Population
Oil content %
Oil content %
Oil content %
Oil content %
Oil content %
Oil content %
Oil content %
Oil content %
Oil content %
Supplementary Figure 2
Frequency distribution of oil content trait in eight bi-parental populations as observed in different environments
Oil content %
