# Supplementary material for: QTL Landscape for Oil Content in Brassica juncea: Analysis in Multiple Bi-Parental Populations in High and “0” Erucic Background
Source: Front Plant Sci. 2018 Oct 16;9:1448. doi: 10.3389/fpls.2018.01448 (PMC6198181; doi:10.3389/fpls.2018.01448)
Supplement: Supplementary file 8 [file Presentation_3.PPTX]

## Slide 1
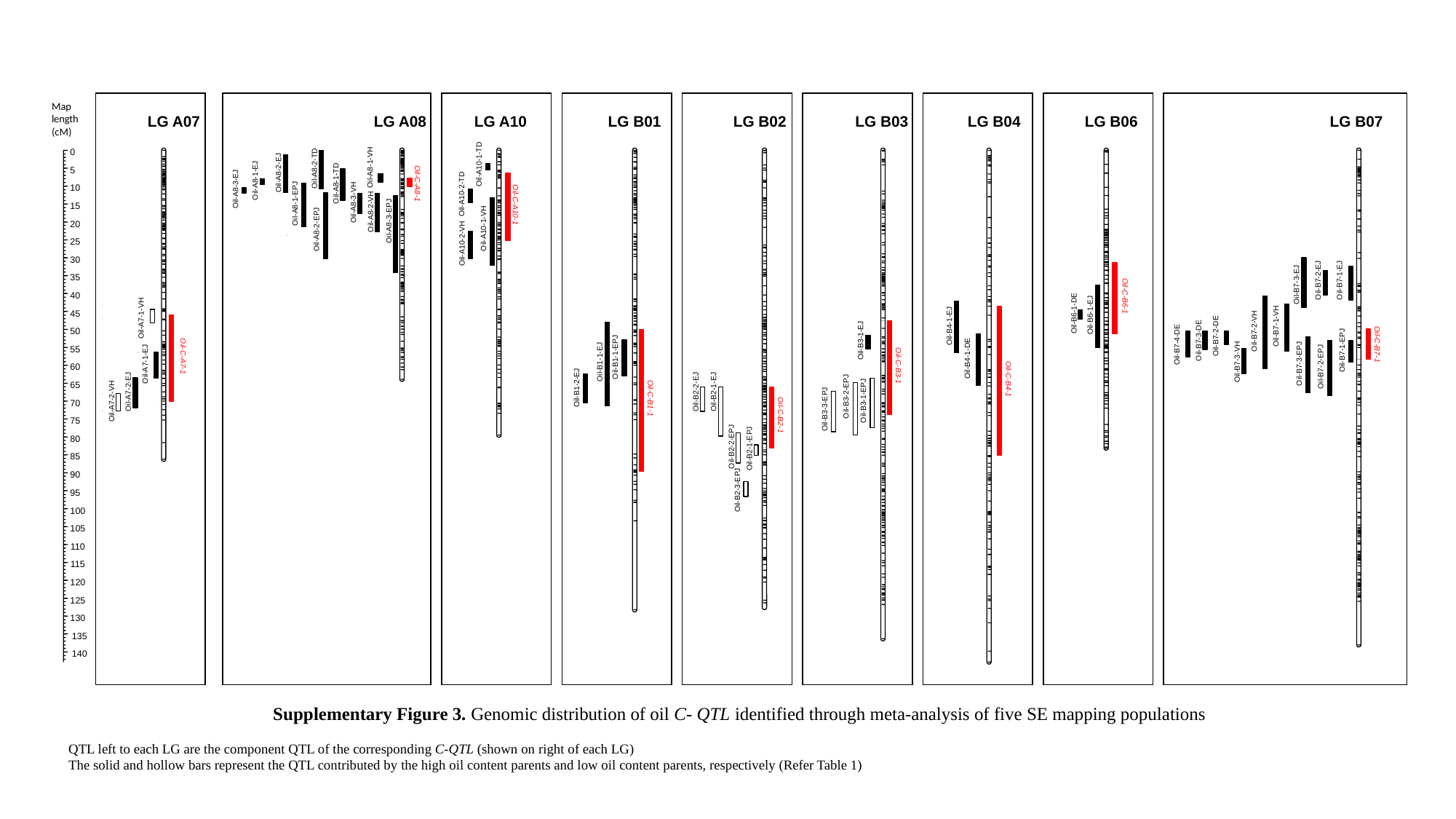

LG A08
Oil-A8-1-VH
Oil-A8-2-TD
Oil-A8-2-EJ
Oil-A8-1-EJ
Oil-A8-1-TD
Oil-C-A8-1
Oil-A8-3-EJ
Oil-A8-3-VH
Oil-A8-1-EPJ
Oil-A8-2-VH
Oil-A8-3-EPJ
Oil-A8-2-EPJ
LG A10
Oil-A10-1-TD
Oil-A10-2-TD
Oil-C-A10-1
Oil-A10-1-VH
Oil-A10-2-VH
LG B01
Oil-B1-1-EPJ
Oil-B1-1-EJ
Oil-B1-2-EJ
Oil-C-B1-1
LG B02
Oil-B2-2-EJ
Oil-B2-1-EJ
Oil-C-B2-1
Oil-B2-2-EPJ
Oil-B2-1-EPJ
Oil-B2-3-EPJ
LG B03
Oil-B3-1-EJ
Oil-C-B3-1
Oil-B3-2-EPJ
Oil-B3-1-EPJ
Oil-B3-3-EPJ
LG B04
Oil-B4-1-EJ
Oil-B4-1-DE
Oil-C-B4-1
LG B06
Oil-C-B6-1
Oil-B6-1-DE
Oil-B6-1-EJ
LG B07
Oil-B7-2-EJ
Oil-B7-1-EJ
Oil-B7-3-EJ
Oil-B7-1-VH
Oil-B7-2-VH
Oil-B7-2-DE
Oil-B7-3-DE
Oil-B7-4-DE
Oil-C-B7-1
Oil-B7-1-EPJ
Oil-B7-3-VH
Oil-B7-3-EPJ
Oil-B7-2-EPJ
Map length (cM)
LG A07
Oil-A7-1-VH
Oil-C-A7-1
Oil-A7-1-EJ
Oil-A7-2-EJ
Oil-A7-2-VH
0
5
10
15
20
25
30
35
40
45
50
55
60
65
70
75
80
85
90
95
100
105
110
115
120
125
130
135
140
Supplementary Figure 3. Genomic distribution of oil C- QTL identified through meta-analysis of five SE mapping populations
QTL left to each LG are the component QTL of the corresponding C-QTL (shown on right of each LG)
The solid and hollow bars represent the QTL contributed by the high oil content parents and low oil content parents, respectively (Refer Table 1)
